# Melting phase change heat transfer in a quasi-petal tube thermal energy storage unit

**DOI:** 10.1371/journal.pone.0246972

**Published:** 2021-03-24

**Authors:** S. A. M. Mehryan, Kaamran Raahemifar, Sayed Reza Ramezani, Ahmad Hajjar, Obai Younis, Pouyan Talebizadeh Sardari, Mohammad Ghalambaz

**Affiliations:** 1 Young Researchers and Elite Club, Yasooj Branch, Islamic Azad University, Yasooj, Iran; 2 College of Information Sciences and Technology (IST), Data Science and Artificial Intelligence Program, Penn State University, State College, Pennsylvania, PA, United States of America; 3 School of Optometry and Vision Science, Faculty of Science, Dept. of Chemical Engineering, Faculty of Engineering, University of Waterloo, Waterloo, ON, Canada; 4 Electrical and Computer Engineering Dept., Sultan Qaboos University, Muscat, Sultanate of Oman; 5 Department of Mechanical Engineering, K. N. Toosi University of Technology, Tehran, Iran; 6 ECAM Lyon, LabECAM, Université de Lyon, Lyon, France; 7 Department of Mechanical Engineering, College of Engineering at Wadi Addwaser, Prince Sattam Bin Abdulaziz University, Al-Kharj, KSA; 8 Department of Mechanical Engineering, Faculty of Engineering, University of Khartoum, Khartoum, Sudan; 9 Faculty of Engineering, The University of Nottingham, University Park, United Kingdom; 10 Metamaterials for Mechanical, Biomechanical and Multiphysical Applications Research Group, Ton Duc Thang University, Ho Chi Minh City, Vietnam; 11 Faculty of Applied Sciences, Ton Duc Thang University, Ho Chi Minh City, Vietnam; Universite des Sciences et de la Technologie Houari Boumediene, ALGERIA

## Abstract

In the present study, the thermal energy storage of a hot petal tube inside a shell-tube type Thermal Energy Storage (TES) unit was addressed. The shell is filled with the capric acid Phase Change Material (PCM) and absorbs the heat from a hot U-tube petal. The governing equations for the natural convection flow of molten PCM and phase change heat transfer were introduced by using the enthalpy-porosity approach. An automatic adaptive mesh scheme was used to track the melting interface. The accuracy and convergence of numerical computations were also controlled by a free step Backward Differentiation Formula. The modeling results were compared with previous experimental data. It was found that the present adaptive mesh approach can adequately the melting heat transfer, and an excellent agreement was found with available literature. The effect of geometrical designs of the petal tube was investigated on the melting response of the thermal energy storage unit. The phase change behavior was analyzed by using temperature distribution contours. The results showed that petal tubes could notably increase the melting rate in the TES unit compared to a typical circular tube. Besides, the more the petal numbers, the better the heat transfer. Using a petal tube could increase the charging power by 44% compared to a circular tube. The placement angle of the tubes is another important design factor which should be selected carefully. For instance, vertical placement of tubes could improve the charging power by 300% compared to a case with the tubes’ horizontal placement.

## 1. Introduction

The energy demand has been increased significantly in recent decades due to the dramatic increase in the population in addition to technological and industrial developments. These advancements have posed a challenge to clean energy production with a justified economic value and not harmful to the environment. Solar and wind energy are perfect examples of sustainable sources of clean energy. One of the biggest challenges to these types of renewable energy is the possibility of storing energy.

One of the well-known energy storage systems is the Latent Heat Thermal Storage System (LHTES). In such storage systems, latent heat is stored by using Phase Change Materials (PCMs). PCMs are known for their great capability of absorbing and releasing thermal energy. Hence in the last two decades, they have been used in engineering applications extensively. These applications include- but are not limited to- heat and tube heat exchangers [1–4], Thermal energy storage systems [5–11], and electronic devices cooling [12–17].

The PCMs usually suffer from poor thermal conductivity, and hence, in the energy storage containers, the natural convection plays a key role in transferring heat to and from PCM. When the PCM phase change to liquid, the natural convection flows could occur in the molten region. Such natural convection flow can contribute to heat transfer and improve the melting rate.

Considering the free convection flows, several studies have addressed the natural convection in enclosures in the literature. Ishak et al. [18] reported a numerical simulation of natural convection and entropy generation in a square cavity, containing nanofluids and subjected to partial heating. In their study, they mainly focused on the impact of cavity wall thickness. They concluded that the wall thicknesses as well as the wall thermal conductivity are essential parameters in controlling and optimizing heat transfer and Bejan number. Natural convection in a finned annulus, being horizontally mounted, was examined by Nada et al. [19]. The authors surveyed the influence of several fin parameters, such as geometry and find distribution. For all studied cases, it was noticed that heat transfer was directly proportional to fins number and width. Among different studied fins geometry, the longitudinal fin was reported to achieve the highest heat transfer rate. Dutta and Biswas [20] numerically analyzed the natural convection and entropy generation in a porous quadrantal cavity. In their study, the authors focused on the influence of Rayleigh and Darcy numbers. They concluded that the irreversibility caused by fluid friction was dominating for high Rayleigh and Darcy numbers. The natural convection of Cu-water nanofluid in a triangular cavity having semicircular lower wall was numerically investigated by Dogonchi et al. [21]. All cavity walls were cooled, while the semicircular lower wall was heated. They succeed in presenting correlations between the average Nusselt number and Rayleigh number, Hartman number, and the volumetric fraction of Cu nanoparticles. The literature works show that the geometry and size of the enclosure could significantly affect the free convection and heat transfer improvement.

The circular enclosures are one of the typical containers for storing liquid and PCMs. In this regard, various annulus geometries have been surveyed in literature. For instance, the free convection has been investigated in the following geometries: a square channel containing a circular cylinder [22], a square cavity with an inner body placed in the cavity center [23], a cavity with heated circular and square cylinders [24], and a rectangular cavity containing an oval-shaped heat source [25]. Mahmoodi and Sebdani [26] explored natural convection in a square cavity filled with nanofluid and contains an adiabatic square at the center. They concluded that the heat transfer rate was inversely proportional to the size of the square for relatively low Rayleigh numbers, while an opposite behavior was noticed for high Rayleigh numbers. The literature results show that the shape and size of the inner obstacle or heated pipe could significantly impact the enclosure’s thermal behavior.

The annulus geometries are of interest for solar collectors, shell and tube heat exchangers, and energy storage units. Thus, irregular shape inner tubes, such as sinusoidal cylinders, have attracted the attention of researchers. Employing a wavy or sinusoidal cylinder can increase the heat transfer surface at the tube side and also influence the hydrodynamic and flow resistance of convection flows. Nabavizadeh et al. [27] undertook numerical simulation of free convection in a square cavity with an inner sinusoidal cylinder. Their results showed that the amplitude or number of undulations significantly influences the thermal and flow field. Cho et al. [28] explored the influence of two elliptic-shaped cylinders having different aspect ratios on the natural convection in a square cavity. The authors reported an increment of 3.9% on average Nusselt number for high Rayleigh number cases in comparison with two circular cylinders. Roslan et al. [29] presented a numerical simulation of free convection in a cavity with an inner cylindrical heat source with sinusoidal heating. They noticed that heat transfer is significantly affected by source temperature signal oscillations. These mentioned studies have analyzed the natural convection heat transfer with no phase change. Thus, it was assumed the working fluid is entirely at the liquid/gas state.

Regarding the natural convection and heat transfer, Gao et al. [30] visualized paraffin melting in a spherical enclosure. The influence of spherical container size, PCM filling ratio, heating, and the initial temperature was studied. The authors proposed a correlation between non-dimensional melting time and liquid fraction. Gortych et al. [31] experimentally and theoretically studied the solidification of PCM in a horizontally mounted annular cavity. The inner cylinder wall was marinated at a temperature less than the solidification temperature. The model developed by the authors was able to capture the influence of various non-dimensional parameters on the solidification process of PCM. The influence of fin material on the melting behavior of PCM in a rectangular cavity was analyzed by Tian et al. [32]. A right wall of the cavity was heated while the other walls were insulated. It was concluded that adding fins resulted in reducing both melting time and capacity of energy-storing per mass.

Farsani et al. [33] attempted to enhance melting and heat transfer characteristics of gallium in a rectangular cavity. Both upper and lower cavity walls were insulted, while the left and right walls were maintained at cold and hot temperatures, respectively. The baffle resulted in a significant improvement in the melting process of gallium. Haddad and Iachachene [34] undertook numerical simulation of organic PCM melting in a trapezoidal enclosure with a wavy bottom wall. All cavity walls were insulted, except the wavy wall, which was maintained at a hot temperature. It was reported that increasing temperature difference resulted in a favorable effect that is much higher than the effect caused by increasing wave amplitude.

The nano-enhanced phase change material (NePCM) are stable PCMs containing nanoparticles with improved thermal conductivity. Lachachene et al. [35] explored the influence of orientation and nanoparticles on PCM melting in a trapezoidal enclosure. The inclined vertical walls were differentially heated, while the lower and upper walls were adiabatic. In general, it was noticed that both effects were favorable; however, for NePCM thermal conductivity enhancement should be> 80%. Sheikholeslami et al. [36] improved the melting process of PCM in a square cylindrical cavity by introducing fins and nanoparticles. The cavity contained an inner cylinder and fins. They concluded that using long fins could improve the melting time. The advantage and properties of NePCM have been further investigated in [37, 38].

The cylindrical thermal energy storage units filled with PCMs have also been investigated in the literature. Li et al. [39] attempted to accelerate PCM’s solidification in an energy storage enclosure by using nanoparticles and an inner sinusoidal cold cylinder. They concluded that the reduction in internal cylinder amplitude resulted in extending solidification time while increasing the volumetric fraction of NEPCM had a favorable impact. Siyabi et al. [40] investigated the impact of the inclination angle of the PCM thermal energy storage system on the heat transfer rate and thermal behavior on the system. Three different inclination angles were considered 0^o^, 45^o^, and 90^o^. Their results indicated that the angle of 45^o^ could lead to the fastest melting rate in comparison to other inclination angles. Ebadi et al. [41] experimentally researched using cooper wire mesh with two different porosities inside a thermal energy storage system. The authors observed a reduction in the charging time and the rate of stored energy due to the higher effective thermal conductivity. Pourakabar and Darzib [42] enhanced the phase change of PCMs in cylindrical containers. They examined nine cases that differ in shell shape and arrangements. It was reported that the arrangement of four tubes in the diamond array resulted in the shortest melting time. Moreover, the usage of metal foam led to 92% and 94% increment in melting and solidification rates, respectively.

Mousavi et al. [43] numerically analyzed melting NePCM in a cylindrical thermal storage system contains horizontal fins. The influence of various nano-PCM and the number of fins was investigated. They noticed that the optimum combination that resulted in a 28% melting time reduction was to use 5% of Al2O3 nano-PCM and three fins. Mostafavi et al. [44] modeled the energy storage in a cylindrical PCM energy storage unit, containing transverse and longitudinal fins. The authors reported that even smaller fins would considerably contribute to magnifying heat transfer.

The cylindrical energy storage units are widely used in thermal energy storage designs, and thus, various aspects of convection heat transfer and thermal energy storage in these enclosures have been investigated in the literature. The literature results highlight that the shape and arrangement of inner tubes could notably change the heat transfer rate and energy storage performance of an energy storage unit. However, the impact of petal tube geometry and arrangements on the heat transfer and energy storage rate of cylindrical enclosures have not been investigated yet. The present study aims to address the impact of the petal-tubes’ arrangement and geometry on the thermal behavior of cylindrical thermal energy storage units for the first time.

## 2. Methodology

### 2.1. Mathematical model

**Fig 1:**
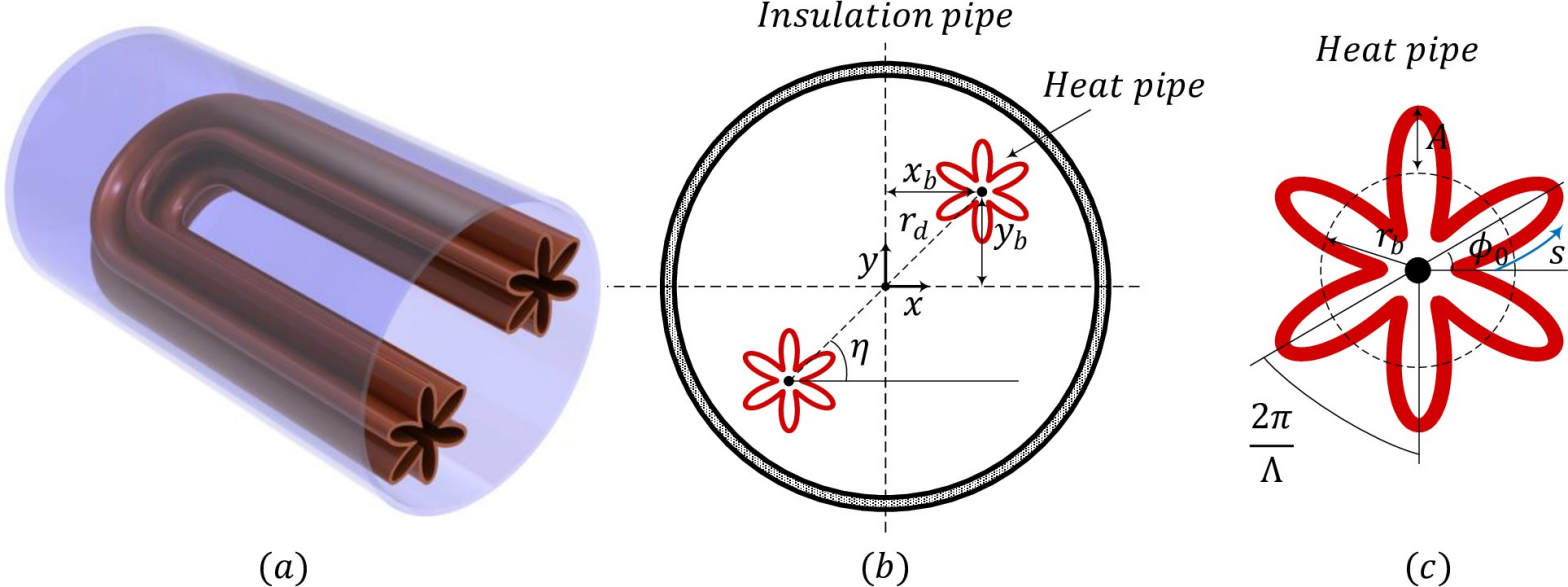
The configuration of the problem physics.

Fig 1 depicts the schematic configuration of a heat exchanger energy storage system. As the schematic view in Fig 1A illustrates, the system includes a shell and a U-shaped tube containing the hot heat transfer fluid (HTF). As can be seen in Fig 1B, the cross-section of the tube is a quasi-petal and the line passing the centers of the heat pipe with respect to the *x*-axis is *η*. The tube is made of a high thermal conductive material, and its thickness is negligible compared to the radius. The shell of the heat exchanger storage system is well insulated, while the tube has the high and low temperatures of *T*_*h*_ = 42°C and *T*_*c*_ = 22°C during the charging process. The convection heat transfer in the heat pipes side is much stronger than the natural convection heat transfer on the NePCM side. Indeed, phase change materials’ thermal conductivity is much smaller than working fluid in the heat pipe. Thus, a variation of the heat pipe’s geometrical shape could result in negligible impacts on the temperature of the tube walls.

The void space between the U-shaped tube and the shell is occupied with Capric acid as the phase change material (PCM) with a nominal melting point of *T*_*m*_ = 32°C. Capric acid [45] and other fatty acids [46] have been investigated for phase change applications recently. The thermophysical properties of the components of the PCM are listed in Table 1. Herein, the density variations of the PCM during phase change are considered to be zero, and the reference densities are selected to simulate the process. The Boussinesq approximation is valid to model the buoyancy-driven convection of the melted PCM. The radius of the sinusoidal cross-section of the quasi-petal heat pipe is defined by using the following relation:

$$r=r_{b}+A\times \sin\left(\Lambda s\right)$$ (1)

**Table 1:** Thermophysical properties of the Capric acid [54, 55].

Properties	Capric acid	
Specific heat (kJ kg^-1^ K^-1^)	Liquid: 2.4	Solid: 1.9
Density (kg m^-3^)	Liquid: 888	Solid: 1018
Thermal conductivity (Wm^-1^ K^-1^)	Liquid: 0.153	Solid: 0.372
Latent heat (kJ kg^-1^)	152.7	
Kinematic viscosity (m^2^ s^-1^)	3×10^−6^	
Fusion temperature (°C)	32	
Thermal expansion coefficient (K^-1^)	1×10^−3^	

As shown in Fig 1C, *r*_*b*_ and *A* are the radius of the base circle and the sinusoidal amplitude of the quasi-petal heat pipe. Λ is the number of petals, and *s* is the angle of the quasi-petal heat pipe. It is proved that the quasi-petal heat pipe area is not dependent on the *Λ* and *A*.

### 2.2. Governing equations

The governing equations for conservation of mass, flow, and energy in the heat exchenger can be written as [33, 35]:

$$\frac{\partial u}{\partial x}+\frac{\partial v}{\partial y}=0$$ (2)

$$\rho_{PCM,l}\left(\frac{\partial u}{\partial t}+u\frac{\partial u}{\partial x}+v\frac{\partial u}{\partial y}\right)=-\frac{\partial p}{\partial x}+\mu_{PCM,l}\left(\frac{\partial^{2}u}{\partial x^{2}}+\frac{\partial^{2}u}{\partial y^{2}}\right)-f\left(T\right)u$$ (3)

$$\rho_{PCM,l}\left(\frac{\partial v}{\partial t}+u\frac{\partial v}{\partial x}+v\frac{\partial v}{\partial y}\right)=-\frac{\partial p}{\partial y}+\mu_{PCM,l}\left(\frac{\partial^{2}v}{\partial x^{2}}+\frac{\partial^{2}v}{\partial y^{2}}\right)-f\left(T\right)v+{\left(\rho \beta \right)}_{PCM,l}g\left(T-T_{m}\right)$$ (4)

$${\left(\rho C_{p}\right)}_{PCM}\left(\frac{\partial T}{\partial t}+u\frac{\partial T}{\partial x}+v\frac{\partial T}{\partial y}\right)=\frac{\partial }{\partial x}\left(\kappa_{PCM}\frac{\partial T}{\partial x}\right)+\frac{\partial }{\partial y}\left(\kappa_{PCM}\frac{\partial T}{\partial y}\right)-\rho_{PCM,l}h_{sf}\frac{\partial \chi \left(T\right)}{\partial t}$$ (5)

Eq (2) denotes the continuity equation while Eqs (3) and (4) are the momentum in x and y directions, respectively. In the above equations, u, v, p, T, and χ are the x-velocity, y-velocity, pressure, temperature, and liquid fraction. The symbols μ, ρ, κ, *h*_*sf*_, *C*_p_, and *β* are the dynamic viscosity, density, thermal conductivity, latent heat, heat capacity, and volume expansion, respectively. The subscripts PCM and l indicate the PCM material and liquid state of PCM, respectively. Tm is a reference temperature, which here is the initial temperature. The source term (*ρβ*)_*PCM*,*l*_*g*(*T*−*T*_*m*_) is the buoyancy term that circulates the liquid PCM due to temperature differences. The term of *f*(*T*)*u* and *f*(*T*)*v* are body forces. These terms have been added to the momentum equations to control the velocity. *f*(*T*) is a function of the temperature and reaches high values in cold solid PCM regions and zero in liquid regions, as introduced below. The source term ∂*χ*(*T*)/∂*t* takes into account the thermal energy storage due to phase change.

$$f\left(T\right)=\varepsilon \frac{{\left(1-\chi \left(T\right)\right)}^{2}}{\chi {\left(T\right)}^{3}+\xi }$$ (6)

It is evident this term is zero and infinity for the completely melted and solid zones. *ε*, the mushy zone constant, shows an approximate magnitude of damping in the momentum equations. This constant is set to 10^5^. Likewise, *ξ* is set to a meager value to prevent division by zero at the melted zone. The liquid fraction of the NePCM, which defines based on the temperature is:

$$\chi \left(T\right)=\left\{ \begin{array}{ll} 0 & T<T_{m}-\frac{\delta T}{2}\\ \frac{T-T_{m}}{\delta T}+\frac{1}{2} & T_{m}-\frac{\delta T}{2}<T<T_{m}+\frac{\delta T}{2}\\ 1 & T>T_{m}+\frac{\delta T}{2} \end{array}\right.$$ (7)

which *T*_*m*_ is the melting temperature, and *δT* is the melting temperature window. The density of PCM, as a function of the melt volume fraction, is:

$$\rho_{PCM}\left(T\right)=\rho_{PCM,l}\chi \left(T\right)+\left(1-\chi \left(T\right)\right)\rho_{PCM,s}$$ (8B)

Also, the thermal conductivity of the PCM, as a function of the melt volume fraction, was attained by means of the following weighted relation:

$$\kappa_{PCM}\left(T\right)=\kappa_{PCM,l}\chi \left(T\right)+\left(1-\chi \left(T\right)\right)\kappa_{PCM,s}$$ (11A)

In a similar way,

$${\left(\rho C_{p}\right)}_{PCM}\left(T\right)={\left(\rho C_{p}\right)}_{PCM,l}\chi \left(T\right)+\left(1-\chi \left(T\right)\right){\left(\rho C_{p}\right)}_{PCM,s}$$ (12B)

The total stored energy in the PCM, which is a combination of sensible and latent heat, was attained by performing an integration over the domain of solution:

$$ES=\int_{A}\int_{T_{in}}^{T}\left[\left(1-\chi \left(T\right)\right){\left(\rho C_{p}\right)}_{PCM,s}+\chi \left(T\right){\left(\rho C_{p}\right)}_{PCM,l}\right]dTdA+\int_{A}\chi \left(T\right){\left(\rho h_{sf}\right)}_{PCM}dA$$ (14)

Initially (*t* = 0), the temperatures of the PCM and quasi-petal heat pipe are *T*_*c*_. At *t* = 24s, the temperature of the heat pipe linearly rises so that it reaches *T*_*h*_ at *t* = 25s. As previously mentioned, the outer shell of the system is well insulated. The radius of the shell is at *r*_*s*_ = 20mm, and the surface are of the quasi-petal heat pipe is kept constant at $A_{p}=\pi r_{s}^{2}/20$.

## 3. Numerical approach

### 3.1. Finite element method

According to the equations of NePCM, it is possible to model the melting process by means of the source terms of the momentum and energy conservation. Moreover, the solid and melted regions are split by a mushy zone, and s(T) controls the field of velocity. The fusion temperature and the melting temperature window (*δT*) is employed to discern these three regions. The mushy zone contains melted and not-melted substances, and there is a strong velocity gradient within its porous media. A high-temperature gradient spot could be produced by the source term of energy, −*ρ*_*NeP*,*l*_*h*_*sf*,*NeP*_*ε*_*j*_(∂*η*(*T*)/∂*t*), representing the heat sink inside the mushy zone and bringing the melting area under control. The quality of the grid is very important in the mushy zone since all these powerful gradients should be considered. Thus, in order to accurately study this area, the mesh adaptation technique as a progressive method is employed for the mushy zone. The Galerkin finite element method (FEM) is utilized to approach the non-linear differential equations. Base on this technique, a weak form of the governing equations can be achieved by the transformation of the equations as well as their corresponding boundary and initial conditions. A shape set, ${\left\{a_{z}\right\}}_{z=1}^{M}$, is employed to expand the dependent variables, including. u, v, p, and T.

$$\left(p,T,u,v\right)\approx \overset{M}{\underset{z=1}{\sum }}\left(p_{z},T_{z},u_{z},v_{z},\right)a_{z}\left(x,y\right)$$ (17)

By imposing Galerkin FEM on the governing equation, a series of residual functions can be established for all nodes of the discretized domain as follow:

$$Q_{m}^{1}\approx \overset{M}{\underset{z=1}{\sum }}u_{z}\int \frac{\partial a_{z}}{\partial x}a_{m}dxdy+\overset{M}{\underset{z=1}{\sum }}v_{z}\int \frac{\partial a_{z}}{\partial y}a_{m}dxdy$$ (18A)

$$\begin{array}{l} Q_{m}^{2}\approx \rho_{NePCM,l}\sum_{z=1}^{M}u_{z}\int \frac{\partial a_{z}}{\partial t}a_{m}dxdy+\rho_{NePCM,l}\sum_{z=1}^{M}u_{z}\int \left[\left(\sum_{z=1}^{M}u_{z}a_{z}\right)\frac{\partial a_{z}}{\partial x}+\left(\sum_{z=1}^{M}v_{z}a_{z}\right)\frac{\partial a_{z}}{\partial y}\right]a_{m}dxdy\\ +\sum_{z=1}^{M}\int \left(-\sum_{z=1}^{M}p_{z}a_{z}\right)\frac{\partial a_{z}}{\partial x}a_{m}dxdy+\mu_{NePCM}\sum_{z=1}^{M}u_{z}\int \frac{\partial a_{z}}{\partial x}\frac{\partial a_{m}}{\partial x}dxdy\\ +\mu_{NePCM}\sum_{z=1}^{M}u_{z}\int \left[\frac{\partial a_{z}}{\partial y}\frac{\partial a_{m}}{\partial y}\right]dxdy-\mu_{NePCM}\int \left(\sum_{z=1}^{M}u_{z}a_{z}\right)a_{m}dxdy-s\left(T\right)\int \left(\sum_{z=1}^{M}u_{z}a_{z}\right)a_{m}dxdy \end{array}$$ (18B)

$$\begin{array}{l} Q_{m}^{3}\approx \rho_{NePCM,l}\sum_{z=1}^{M}v_{z}\int \frac{\partial a_{z}}{\partial t}a_{m}dxdy+\rho_{NePCM,l}\sum_{z=1}^{M}v_{z}\int \left[\left(\sum_{z=1}^{M}u_{z}a_{z}\right)\frac{\partial a_{z}}{\partial x}+\left(\sum_{z=1}^{M}v_{z}a_{z}\right)\frac{\partial a_{z}}{\partial y}\right]a_{m}dxdy\\ +\sum_{z=1}^{M}\int \left(-\sum_{jz1}^{M}p_{z}a_{z}\right)\frac{\partial a_{z}}{\partial y}a_{m}dxdy+\mu_{NePCM}\sum_{z=1}^{M}v_{z}\int \frac{\partial a_{z}}{\partial x}\frac{\partial a_{m}}{\partial x}dxdy\\ +\mu_{NePCM}\sum_{z=1}^{M}v_{z}\int \left[\frac{\partial a_{z}}{\partial y}\frac{\partial a_{m}}{\partial y}\right]dxdy-\mu_{NePCM}\int \left(\sum_{z=1}^{M}v_{z}a_{z}\right)a_{m}dxdy\\ -s\left(T\right)\int \left(\sum_{z=1}^{M}v_{z}a_{z}\right)a_{m}dxdy+{\left(\rho \beta \right)}_{NePCM,l}g\left(\int \left(\sum_{z=1}^{M}T_{z}a_{z}\right)a_{m}dxdy-T_{fu}\right) \end{array}$$ (18C)

$$\begin{array}{l} Q_{m}^{4}\approx {\left(\rho C_{p}\right)}_{NePCM}\sum_{z=1}^{M}T_{z}\int \frac{\partial a_{z}}{\partial t}a_{m}dxdy\\ +{\left(\rho C_{p}\right)}_{\mathrm{NePCM}}\sum_{z=1}^{M}T_{z}\int \left[\left(\sum_{z=1}^{N}u_{z}a_{z}\right)\frac{\partial a_{z}}{\partial x}+\left(\sum_{z=1}^{M}v_{z}a_{z}\right)\frac{\partial a_{z}}{\partial y}\right]a_{m}dxdy\\ +k_{\mathrm{NePCM}}\sum_{z=1}^{M}T_{z}\int \left[\frac{\partial a_{z}}{\partial x}\frac{\partial a_{m}}{\partial x}+\frac{\partial a_{z}}{\partial y}\frac{\partial a_{m}}{\partial y}\right]dxdy-{\left(\rho h\right)}_{NePCM}\sum_{z=1}^{M}\frac{\partial \chi (T)}{\partial T}\int \frac{\partial a_{z}}{\partial t}a_{m}dxdy \end{array}$$ (18D)

These residual functions are integrated through the second-order Gaussian-quadrature rule. The set of above equation provides algebraic equations for each element that should be solved iteratively for the dependent variable. Regarding the Galerkin finite element method, a comprehensive study is presented in [47, 48]. Here, an axillary phase field was introduced slightly wider than the phase change region. This axillary phase field was used as a mesh adaptation control. The temperature bond of 3δ*T*/2, instead of *δT*, was adopted for the axillary phase-field (χ_0_). The space inside the axillary phase field was tagged as χ_0_ = 1. Here, χ_0_ can be expressed as:

$$\eta_{0}\left(T\right)=\left\{ \begin{array}{ll} 0 & T\leq T_{fu}-\frac{3}{2}\delta T\\ 1 & T_{fu}-\frac{3}{2}\delta T<T<T_{fu}+\frac{3}{2}\delta T\\ 0 & T\geq T_{fu}+\frac{3}{2}\delta T \end{array}\right.$$ (19)

Such a larger temperature field as a fusion bond brings about a larger zone around the solidification/melting interface and contributes to a smooth transient of mesh in the vicinity of the melting interface. Inside the χ_0_ domain, the mesh size is refined as five-times finer than the typical domain grids. Moreover, when the phase change interface forms inside the adapted region, χ_0 = 1_, the number of elements is sufficient for the computational procedure. Subsequently, a sufficiently large χ_0 = 1_ leads to a reasonable space for the advancement of the melting interface and reduces the requirement of adaptation steps, and consequently, the adaptation cost.

By means of free step Backward Differentiation Formula (BDF), an automatic time step range of 1–2 is considered [49]. The PARallel DIrect SOlver (PARDISO) solver [50–52] with Newton method was utilized to solve the residual equations iteratively. A residual error O(10^−6^) and a Newtonian damping factor of 0.8 were employed to iteratively solve the residual equations of Eq (18).

### 3.2. Grid-independency test

**Fig 2:**
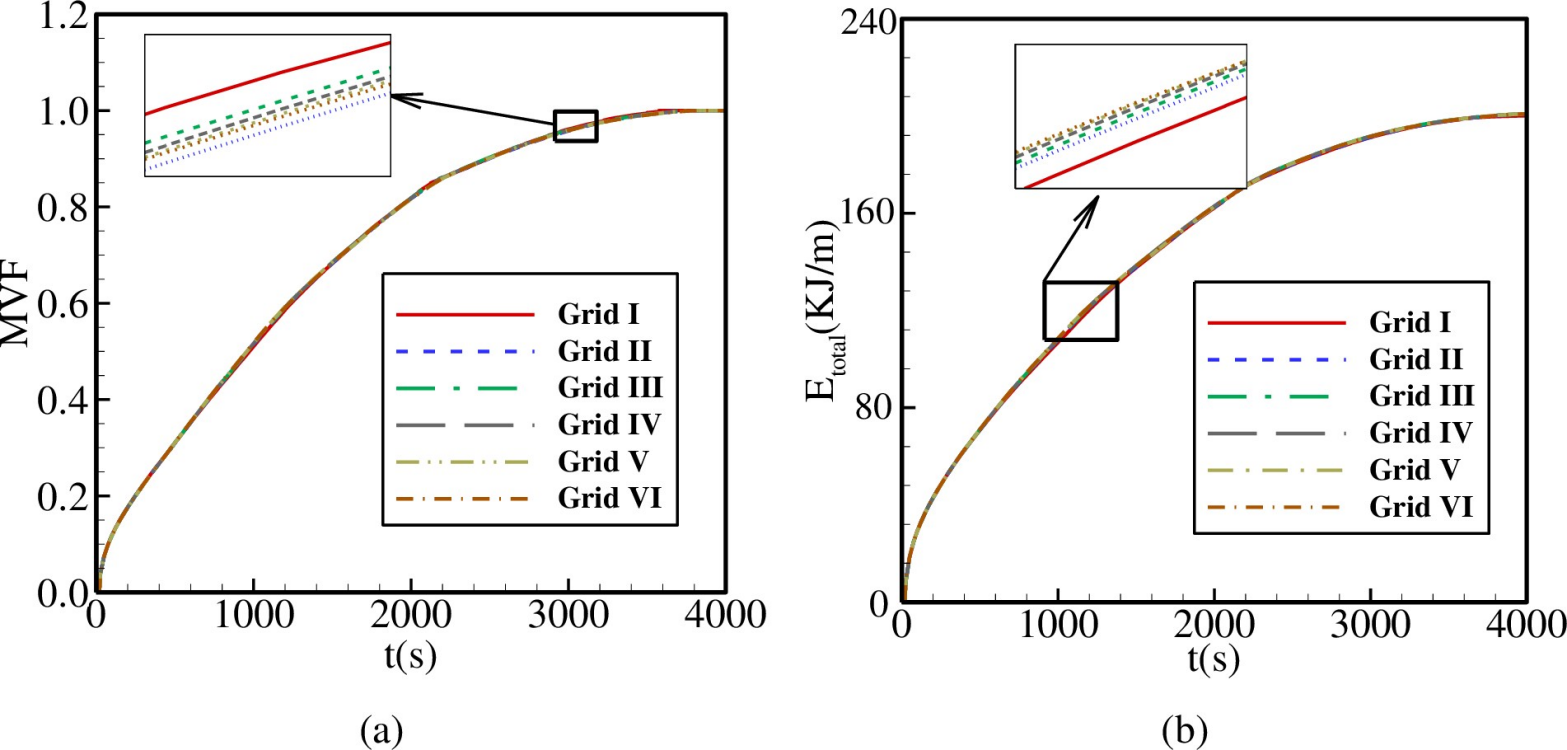
The influence of mesh size on (a): the melt volume fraction (MVF); (b): the total energy stored (E_total_).

**Fig 3:**
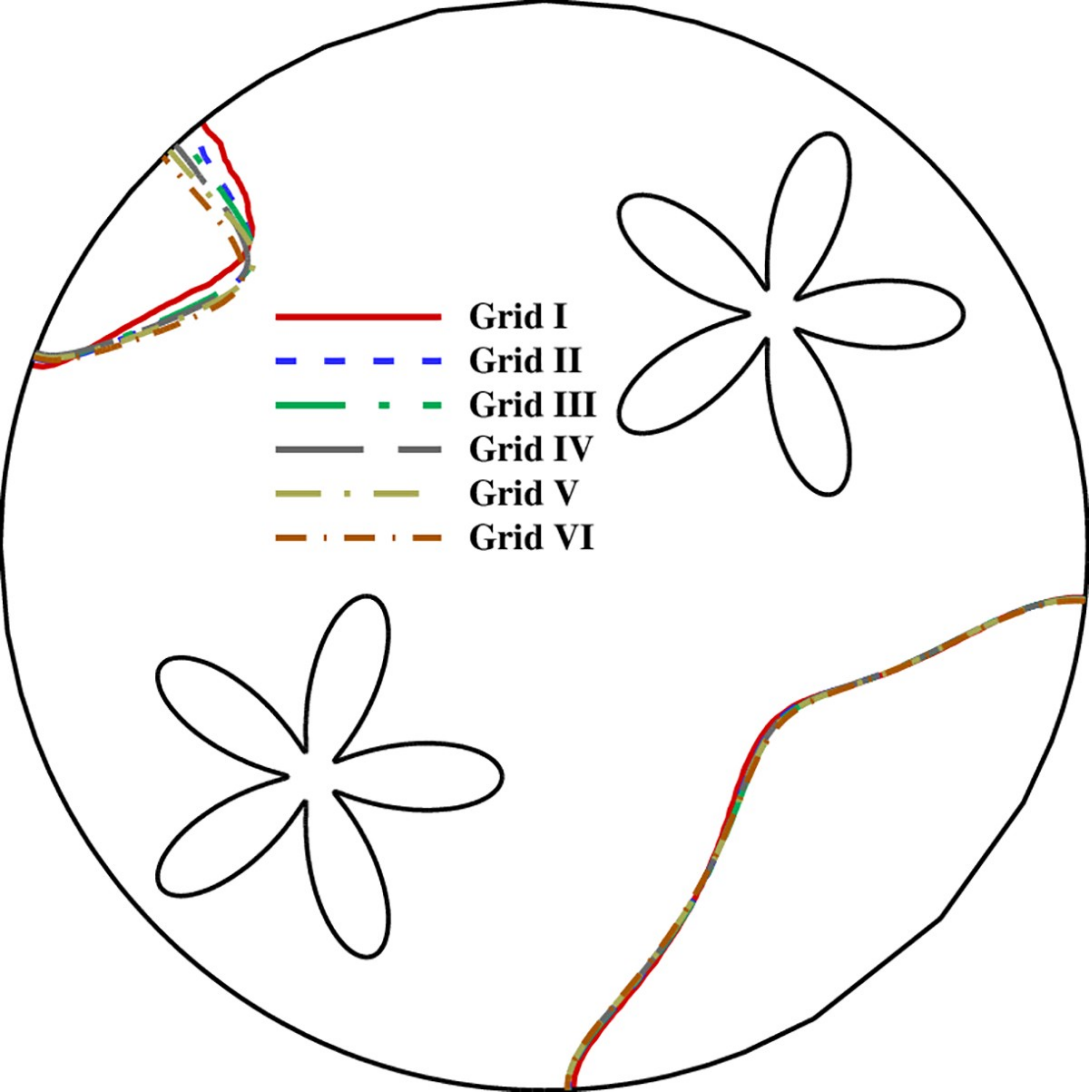
The influence of mesh size on the melting interface at t = 1750s (MVF = 0.75).

**Fig 4:**
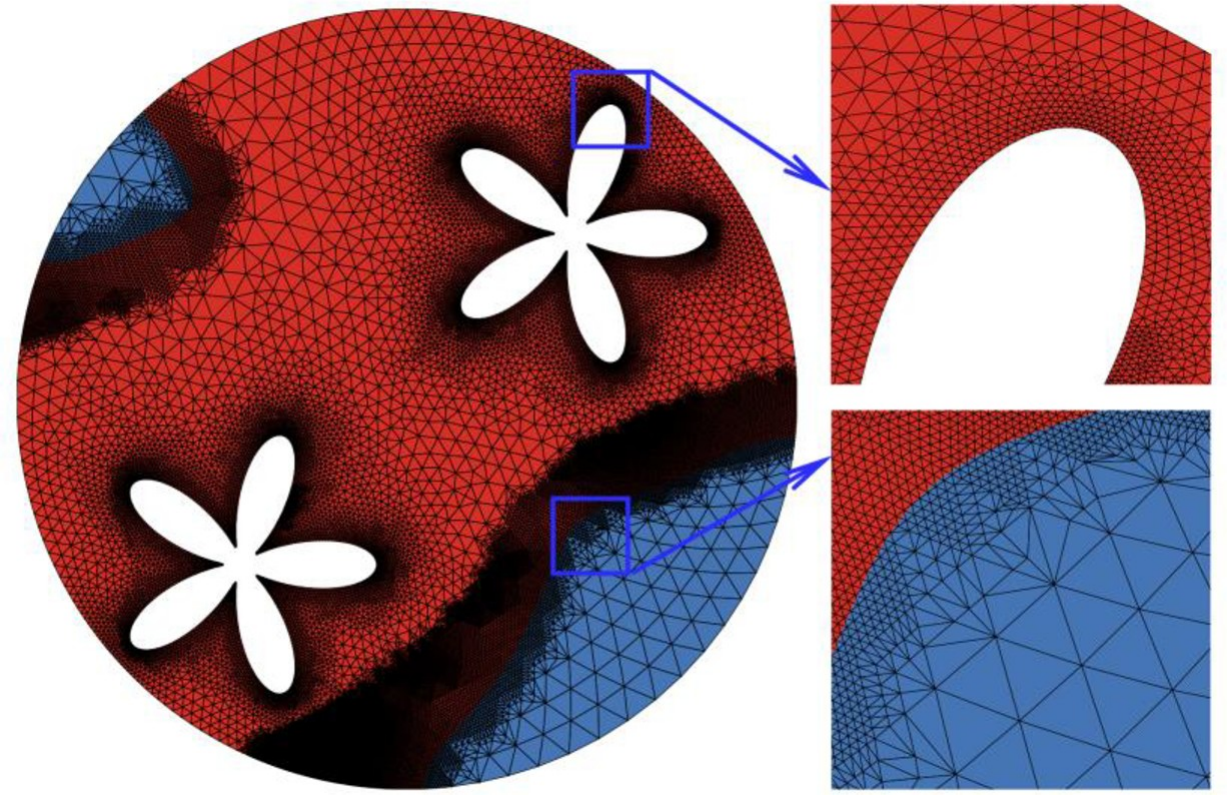
The utilized gird for computations (Grid 5) at t = 1750s (MVF = 0.75).

In order to achieve reliable results, a quality mesh is crucial. For this purpose, the grid-independency is tested through six grids with different numbers where the details are represented in Table 2. The mesh study was performed for *A* = 0.15rs, *Λ* = 5, and *η* = π/4. According to Figs 2 and 3, variables involved in the mesh-independency test are melting volume fraction, stored energy and melting interface. By investigating the graphs for all cases, it is evident that all cases, especially III, IV, and V, are almost accordant with each other. For efficiency in the time and the cost of the numerical calculations, case V is selected. A total number of 28619 (1835) domain (boundary) elements are used in case V. Moreover, Fig 4 illustrates the mesh deformation and the adaptive refinement t = 1750s. A sensible transient is shown in Fig 4 where the blue color is to the solid region. The red color represents the liquid area.

**Table 2:** The details of the utilized grids when *r*_*d*_ = 0.6*r*_*s*_, *A* = 0.15*r*_*s*_, Λ = 5, and *η* = π/4.

Elements number	Examined cases					
	I	II	III	IV	V	VI
**Boundary**	1338	1359	1379	1524	**1835**	2135
**Domain**	7818	13457	17065	24324	**28619**	36431
**Computing time (s)**	1668	5027	6453	11887	**12626**	15286

### 3.3. Validation

The results are validated through the previous experimental and numerical studies, and the accuracy of the numerical modeling is assessed. Initially, in order to verify the present study, the results are compared to the investigation conducted by Kumar et al. [56], where they experimentally examined the interface and melting process of solid-liquid phases using neutron radiography of the flux. As for the boundary condition, they considered the square cavity with its vertical walls exposed to constant heat flux, and other walls were insulated.

**Fig 5:**
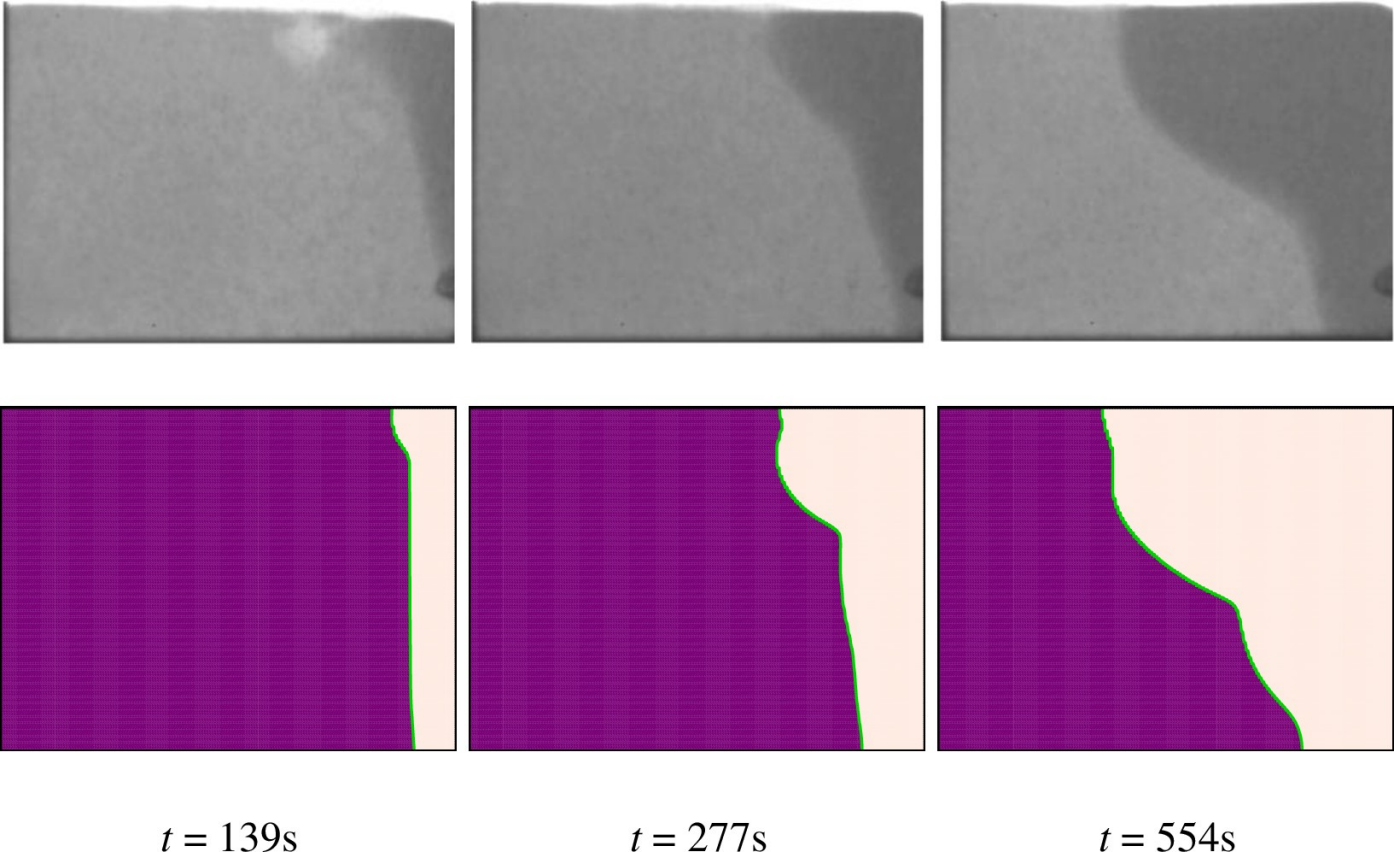
The comparison of experimental and numerical results: First row experiments of Kumar et al. [56], and the second row: The present simulation.

Fig 5 illustrates both the results presented in [56] about the melting front and the corresponding results reported in this study. For element heat flux of 16.3 kW/m2, Ra = 1.4×107, Pr = 0.0236, and Ste = 0.4 the outcomes of four-time steps are shown. The melting front in both studies are exactly alike, and it is clear that the reported results are in good agreement. Moreover, the numerical method used in this study also validated by reported results of [57] in which the melting process of a base PCM (in the absence of nanoparticles) inside a cavity was studied. The vertical walls were exposed to the constant temperature boundary condition, with the left wall and the right wall were hot and cold (T_h_ > T_c_), respectively. The two horizontal walls of the cavity were considered insulated.

**Fig 6:**
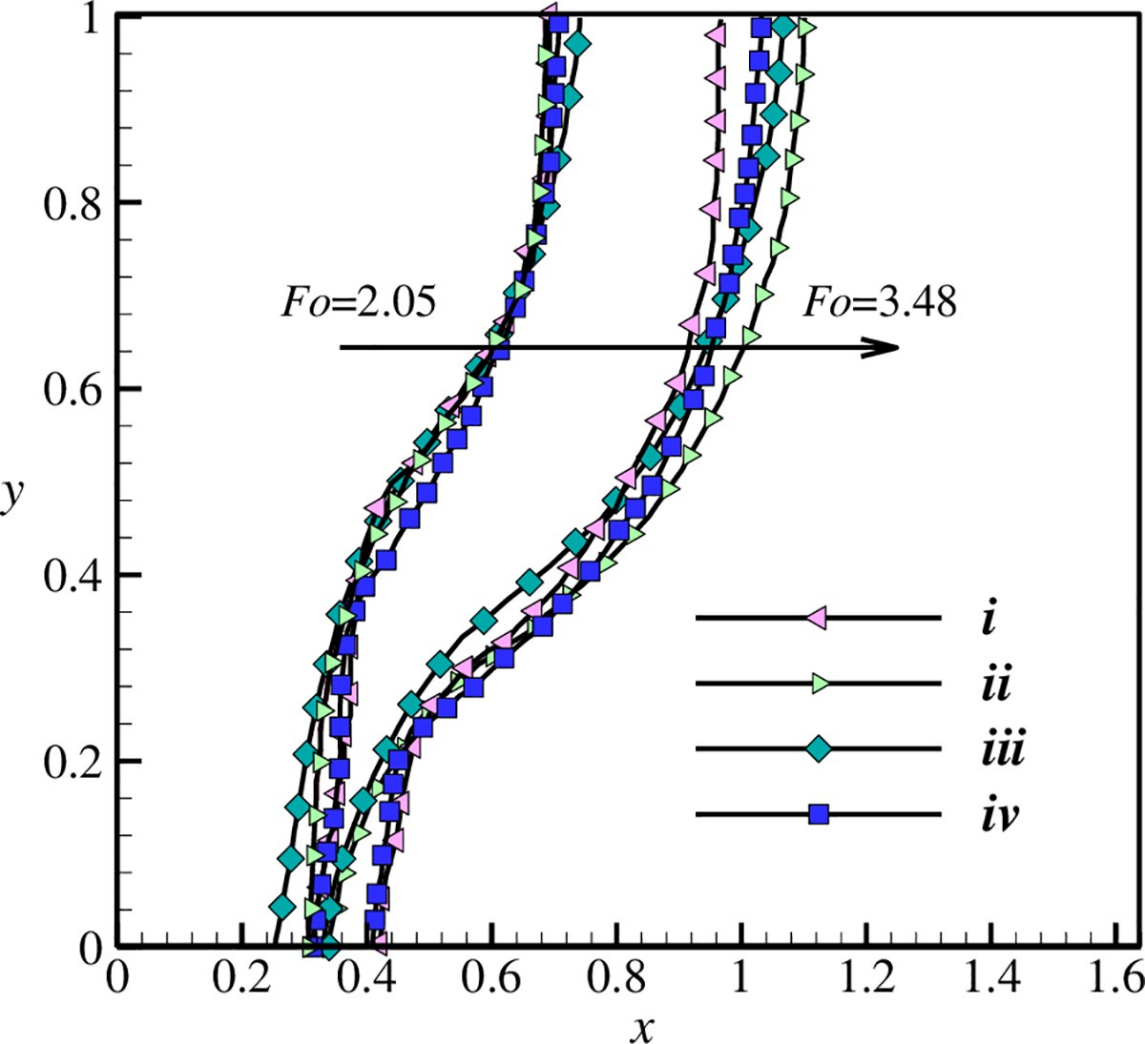
The melting interface simulated in the present study and the literature review of [57]; i: Kashani et al., ii: Gau and Viskanta (experiment); iii: Brent et al., and iv: present work.

**Fig 7:**
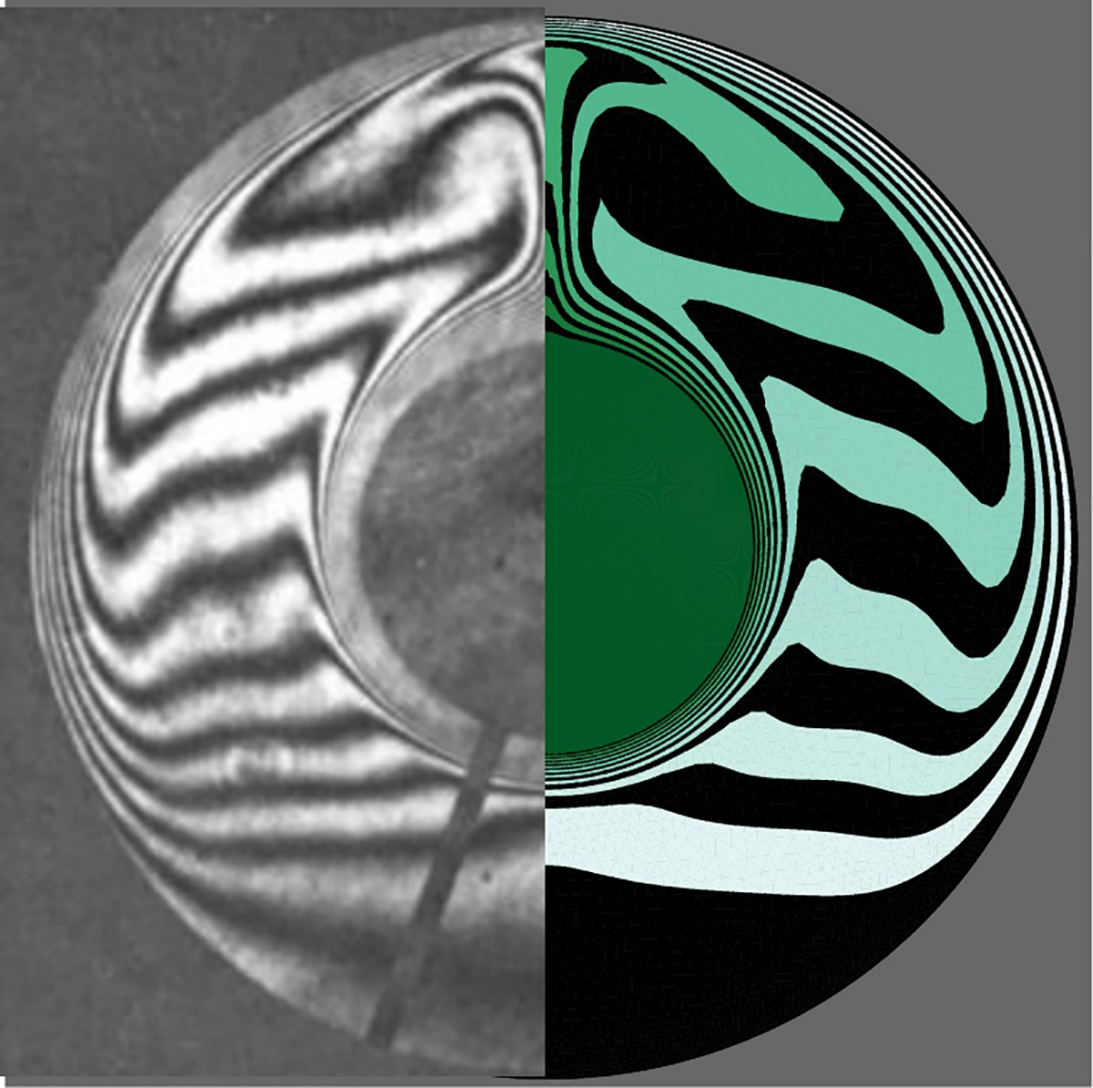
The experimental observations of [53] (right) and the present simulation (left).

The comparison between the results when Pr = 50 and Ra = 1.25×10^5^ is preformed in Fig 6, which indicates both studies are completely in agreement with each other. In order to validate the natural convection heat transfer characteristic of the present study, the research of Kuehn and Goldstein [53] is considered. The heat transfer inside the space of two horizontal cylinders is experimentally investigated in their study. The working fluids of water and air were employed where the gap width (L) to the internal cylinder’ diameter (Di) was L/Di = 0.8. For Ra = 2.33×105 and Pr = 6.19, the isotherm contours are illustrated in Fig 7, where the results are clearly compatible with each other.

## 4. Results and discussion

The parameters can be studied in this work are *r*_*d*_, *A*, Λ, and *η* with the following ranges: 0.4 *r*_*s*_, ≤ *r*_*d*_ ≤ 0.6 *r*_*s*_, 0 ≤ *A* ≤ 0.15 *r*_*s*_, 2 ≤ Λ ≤ 8, and 0 ≤ *η* ≤ π/2. Moreover *ϕ*_0_, i.e., the angle of the first petal with respect to the horizontal direction can be varied based on the number of petals.

**Fig 8:**
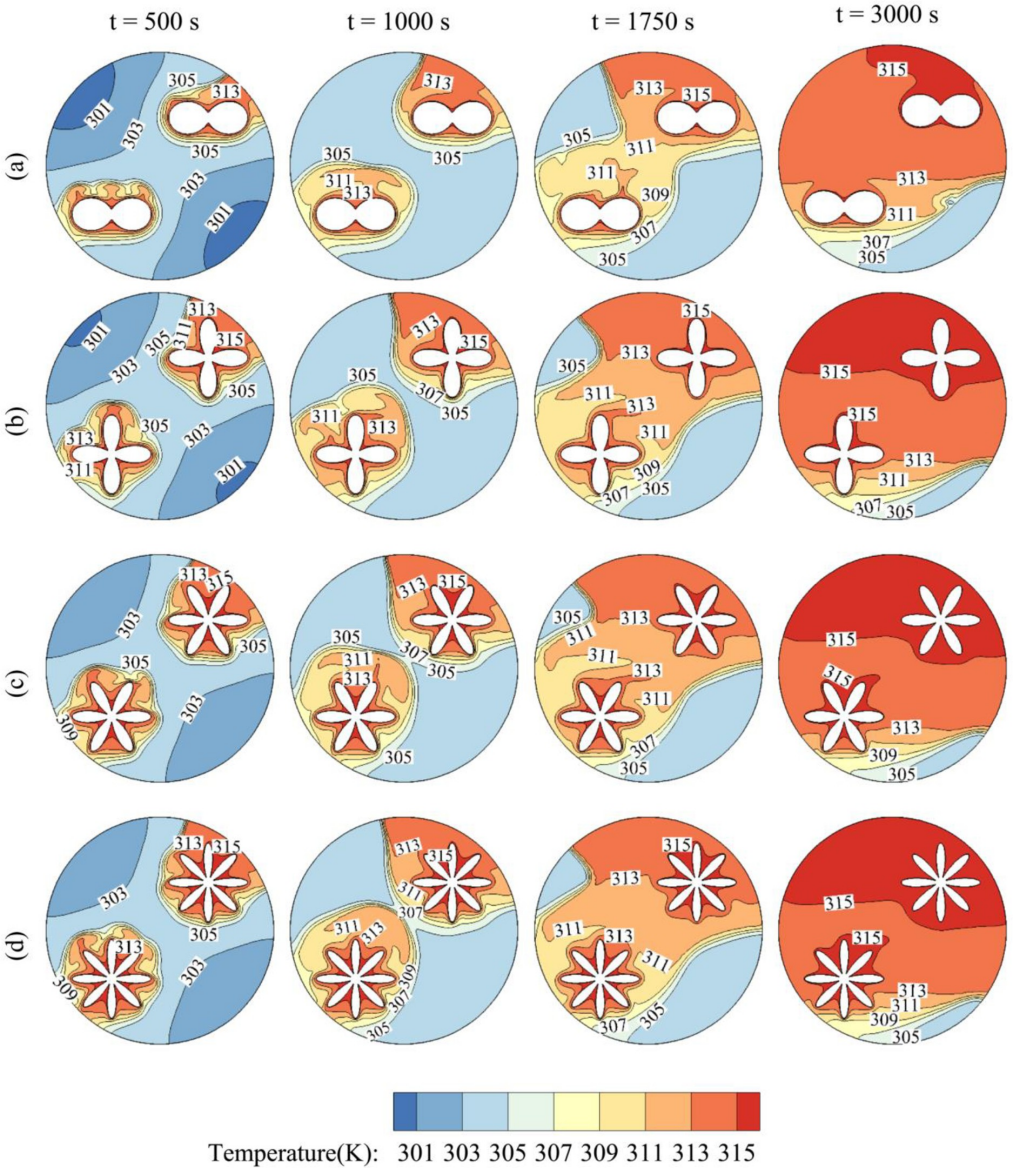
The contours of Temperature and the effects of the number of petals of the quasi-petal heat pipe on contours of Temperature. (a): Λ = 2; (b): Λ = 4; (c): Λ = 6; (d): Λ = 8 when *r*_*d*_ = 0.6 *r*_*s*_, A = 0.15 *r*_*s*_, and η = π/4.

Fig 8 depicts the time evolution of the temperature contours in the exchanger for various values of the number of petals Λ. It should be noted that the fusion temperature of the PCM is T_m_ = 305 k, which means that PCM in the zones where the temperature is higher than T_m_ is melted and in the liquid state, while in the other zones, it remains in the solid-state. In all the cases, at t = 500 s, the PCM is heated only in the close region surrounding the inner tube. As time goes, the heated zone increases in size, and as the PCM melts, it undergoes free convection as the hot melt moves upwards. Finally, at t = 3000 s, most of the PCM in the cavity has melted except in a small region near the bottom, where the convective effects are less significant in that zone. The temperature presents similar contours for all the values of Λ. However, it is clear that the zone of solid PCM is larger in size for lower Λ and is maximum for Λ = 2. In fact, when the number of petals is higher, the contact surface between the inner hot tube and the surrounding PCM is greater, and the heat transfer between the two systems is higher. Nonetheless, it can be seen that for Λ = 6 and Λ = 8, the temperature contours are very similar, indicating that increasing Λ above 6 leads almost to the same result.

**Fig 9:**
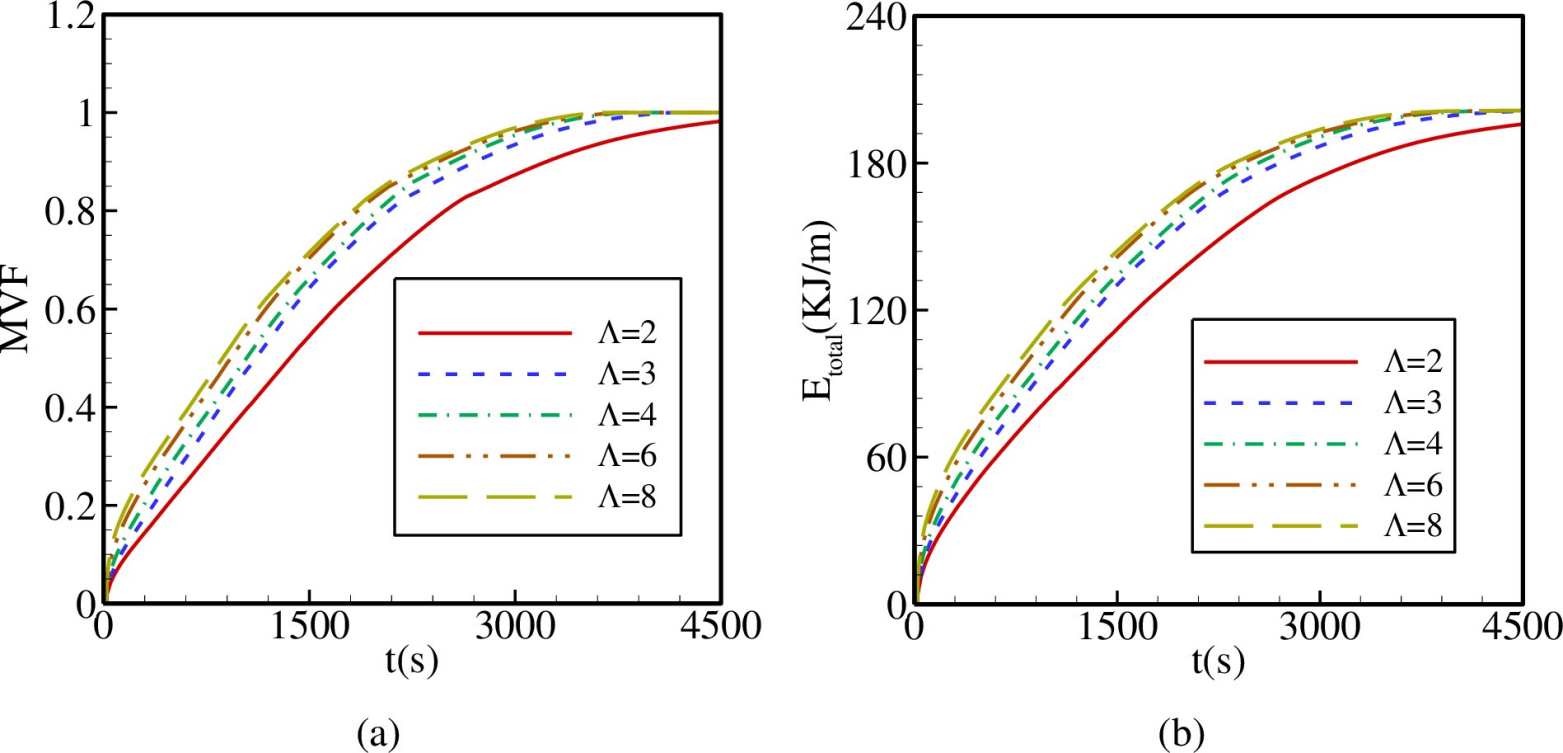
The effects of the number of petals of the quasi-petal heat pipe on (a): the melt volume fraction (MVF); (b): the total energy stored (E_total_) when *r*_*d*_ = 0.6 *r*_*s*_, A = 0.15 *r*_*s*_, and η = π/4.

Fig 9 shows the variations of the PCM melted volume fraction MVF and the total stored energy E_total_ as functions of time for different values of Λ. It is clear that the total melting time, i.e., the time required to reach the value MVF = 1 increases when Λ is reduced and is maximum for Λ = 2. On the other hand, E_total_ is lower throughout time when Λ is decreased and is minimum for Λ = 2. These observations are related to the enhanced heat transfer due to the increase of the contact between the inner hot tube and the surrounding PCM when the number of petals is increased.

**Fig 10:**
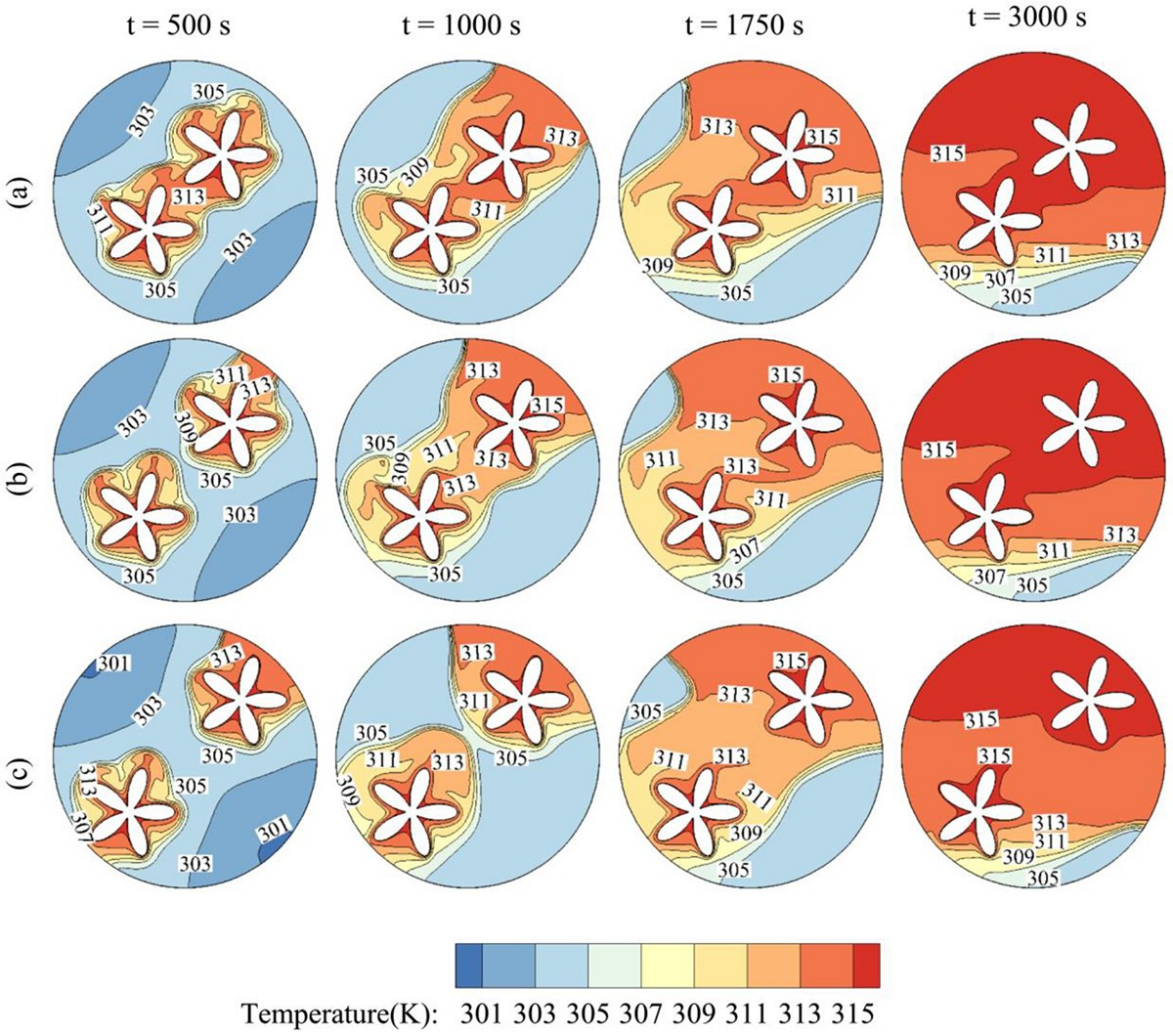
The contours of *r*_*d*_ on contours of Temperature. (a): *r*_*d*_ = 0.4; (b): *r*_*d*_ = 0.5; (c): *r*_*d*_ = 0.6 when A = 0.15 *r*_*s*_, Λ = 5, and η = π/4.

The effect of the radius of the base circle of the inner pipe, *r*_*d*_, on the development of temperature contours with time is illustrated in Fig 10. *r*_*d*_ represents the distance between the two branches of the inner tube, and reducing *r*_*d*_ shifts the two branches of the tube towards the center of the cavity. The main effect of *r*_*d*_ on the temperature contours appears in the initial stages, which depends on the location of the inner tube. At t = 500 s, for low *r*_*d*_, the PCM is mainly heated near the center of the cavity, while for higher *r*_*d*_, heating mainly occurs close to the outer shell. As time goes, the PCM is heated and melts and the temperature contours become similar for all the values of *r*_*d*_. Nonetheless, the temperature in the bottom part of the cavity is slightly higher for higher *r*_*d*_.

**Fig 11:**
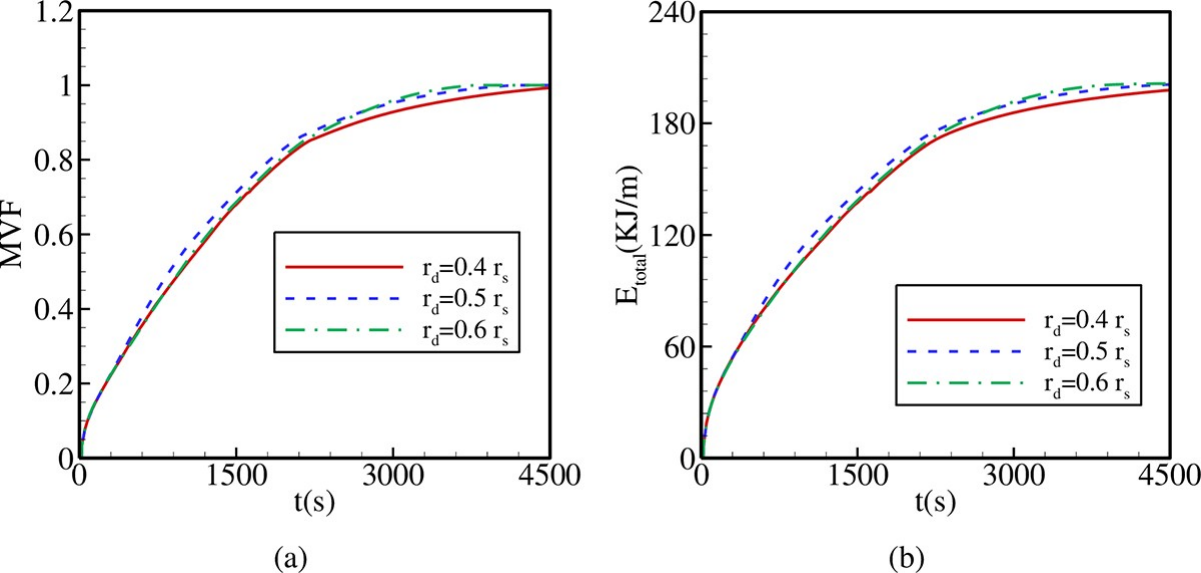
The effects of *r*_*d*_ on (a): the melt volume fraction (MVF); (b): the total energy stored (E_total_) when A = 0.15 *r*_*s*_, Λ = 5, and η = π/4.

The variations of MVF and E_total_ as functions of time are plotted in Fig 11 for different values of *r*_*d*_. Raising *r*_*d*_ slightly increases the melting rate and the value of E_total_. Total PCM melting is reached faster for higher values of *r*_*d*_. Thus, shifting the inner tube away from the center enhances PCM melting, which is due to the spread of heat transfer over a larger zone mainly close to the shell.

**Fig 12:**
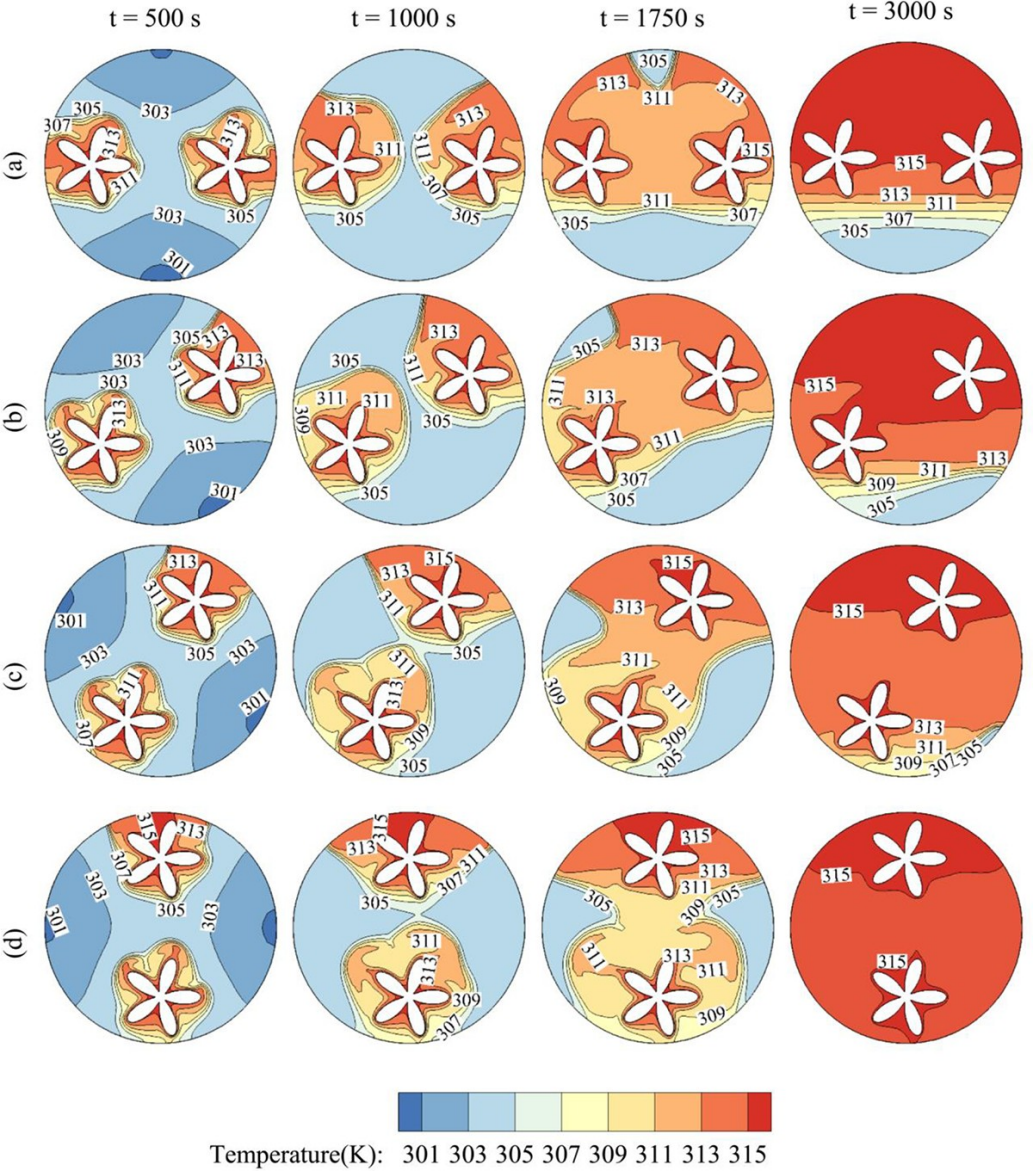
The contours of Temperature and the effects of η on contours of Temperature. (a): η = 0; (b): η = π/6; (c): η = π/3; (d): η = π/2 when *r*_*d*_ = 0.6 *r*_*s*_, A = 0.15 *r*_*s*_, and Λ = 5.

Fig 12 illustrates the impact of the angle of inclination of the inner tube on the time evolution of the temperature contours. η = 0 corresponds to a horizontal tube while η = π/2 corresponds to a vertical 1. It is clear that increasing η increases the overall temperature of the PCM in the cavity and enhances its melting. More particularly, for η = 90, the PCM is totally melted at t = 3000 s, while a large part of the PCM remains in the solid-state in the bottom region for η = 0. In fact, for η = π/2, the PCM is initially melted at the top and bottom of the cavity, and by free convection, the hot melt goes upwards, and heat is transferred over the cavity, leading to full melting. On the other hand, when the tube is horizontal, the PCM is initially melted at the right and left of the cavity, and as time goes, the convective effects are limited to the upper part of the cavity, while the bottom remains relatively cold.

**Fig 13:**
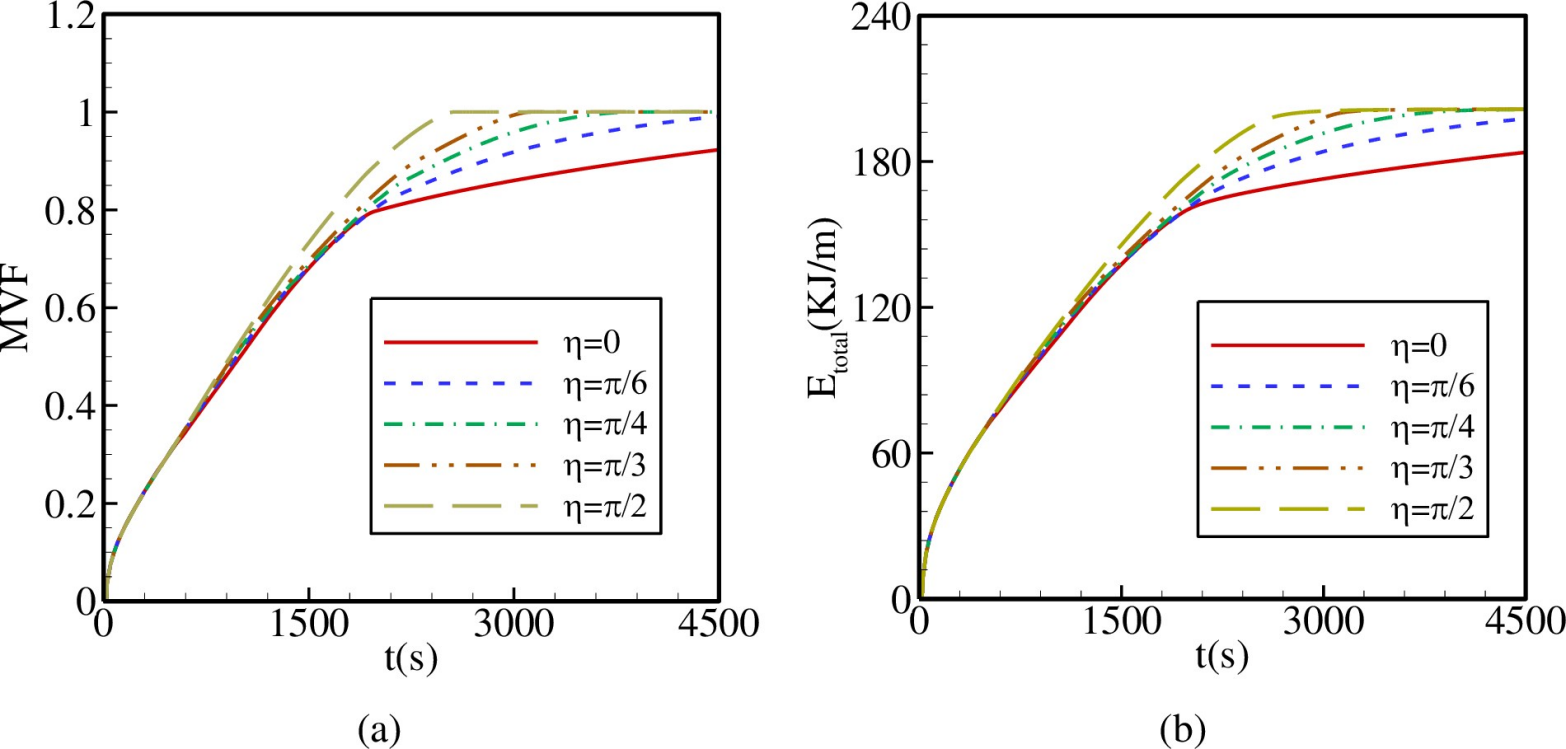
The effects of *η* on (a): the melt volume fraction (MVF); (b): the total energy stored (E_total_) when *r*_*d*_ = 0.6 *r*_*s*_, A = 0.15 *r*_*s*_, and Λ = 5.

The variations of MVF and E_total_ as functions of time are shown in Fig 13 for various values of η. It is evident that raising η increases the melting rate and E_total_. A substantial increase in the total melting time is obtained when η is raised from 0 to π/2. In fact, total melting is obtained after t = 2250 s for η = π/2, while for η = 0, total melting is not attained even after 4500 s. These results are in accordance with the observations of Fig 9, in which it was discussed that placing the inner tube in the vertical position substantially improves the convective heat transfer all over the cavity and enhances PCM melting.

**Fig 14:**
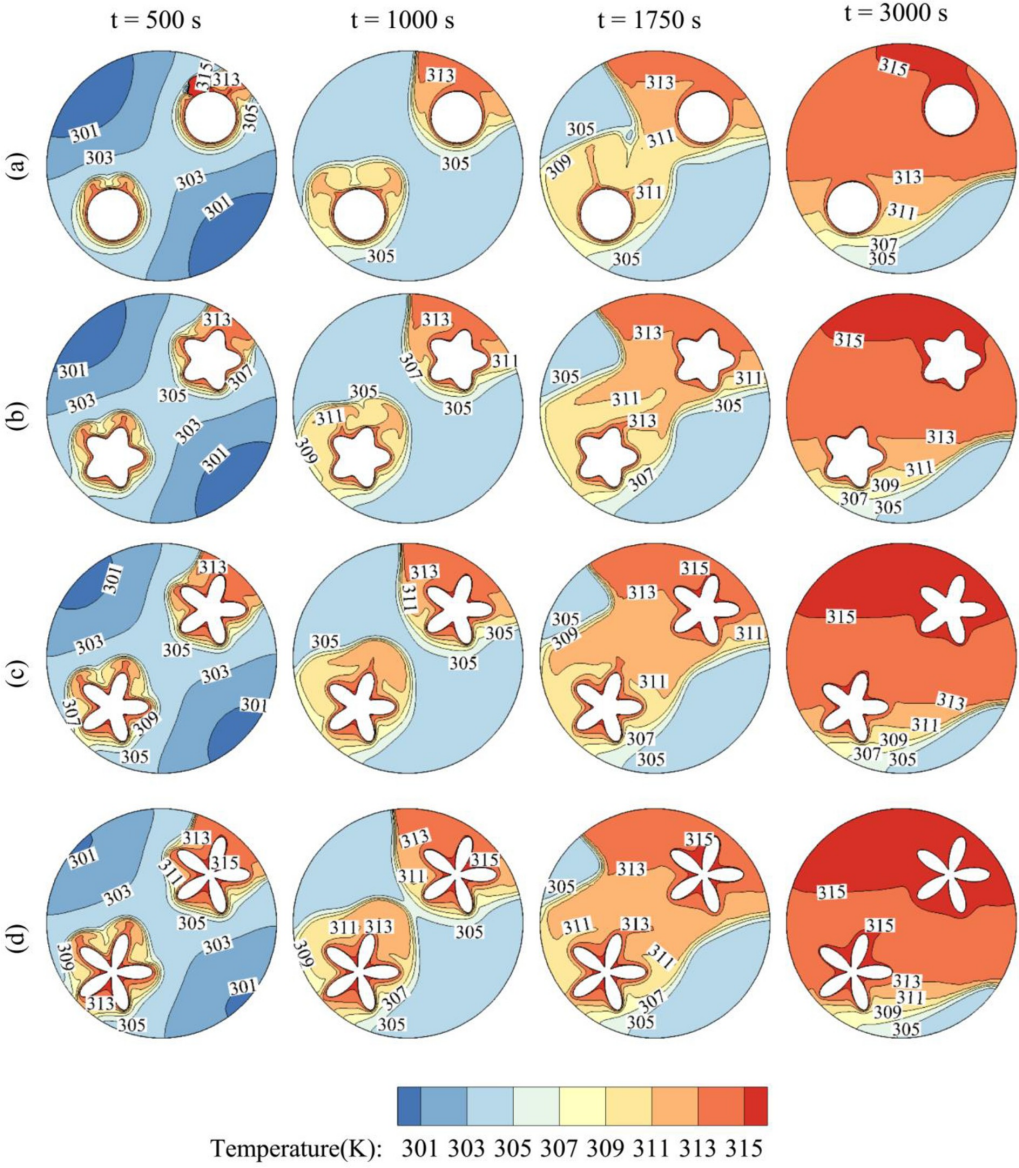
The contours of Temperature and the effects of the sinusoidal amplitude of the quasi-petal heat pipe on contours of Temperature. (a): *A* = 0; (b): *A* = 0.05 *r*_*s*_; (c): A = 0.10 *r*_*s*_; (d): A = 0.15 *r*_*s*_ when *r*_*d*_ = 0.6 *r*_*s*_, Λ = 5, and η = π/4.

The effect of the petal sinusoidal amplitude, A, on the time evolution of the temperature contours is depicted in Fig 14. It can be seen that the temperature of the PCM in the cavity is higher when A is increased. Moreover, the amount of solid PCM is greater when A is reduced and is maximum when the tube has a circular cross-section (A = 0). Indeed, increasing the amplitude of the petals indicates a greater contact between the inner hot tube and the PCM and, consequently, enhances heat transfer.

**Fig 15:**
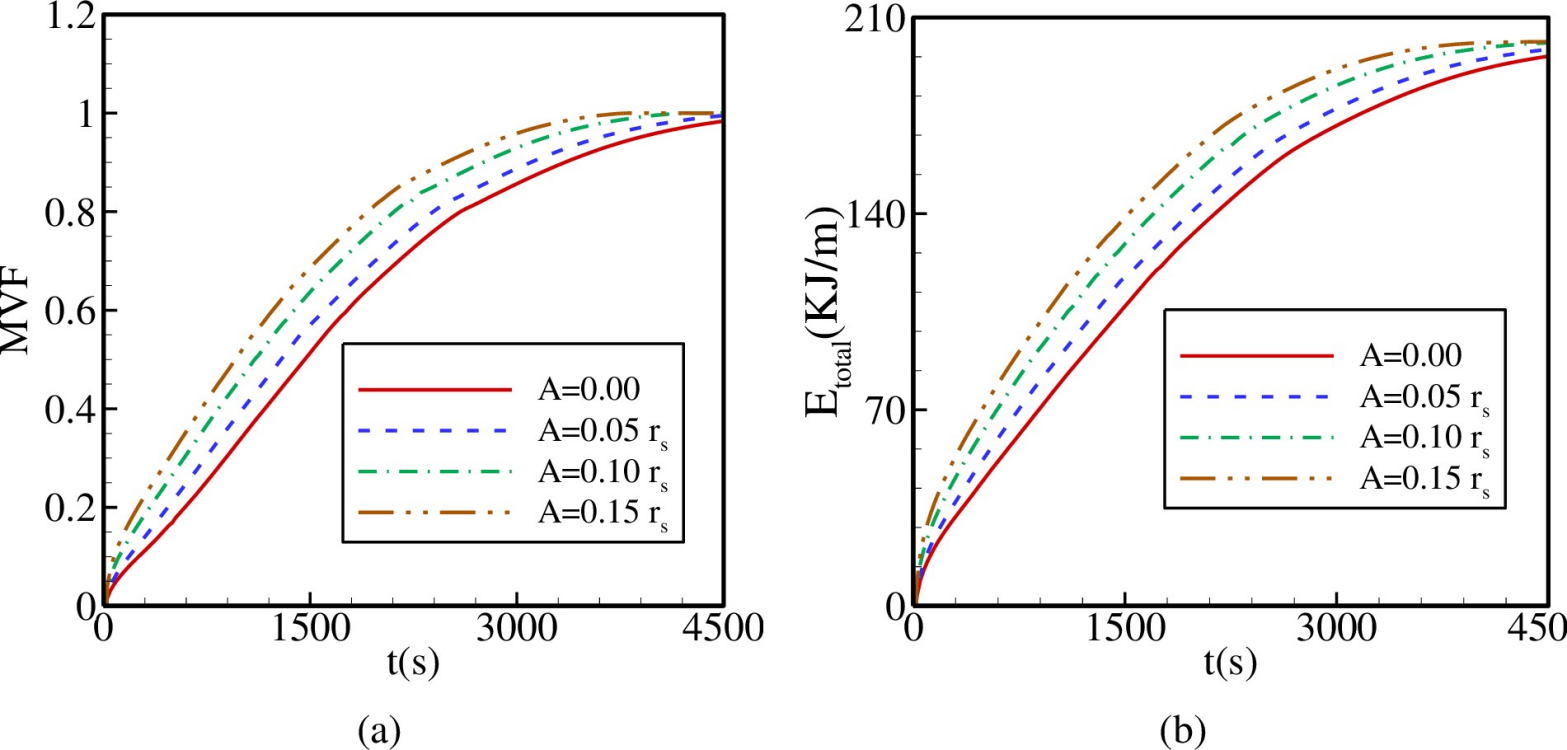
The effects of the sinusoidal amplitude of the quasi-petal heat pipe on (a): the melt volume fraction (MVF); (b): the total energy stored (E_total_) when *r*_*d*_ = 0.6 *r*_*s*_, Λ = 5, and η = π/4.

Fig 15 illustrates the variations of MVF and E_total_ as functions of time for different values of *A*. By increasing the contact area between the hot tube and the PCM and, consequently, heat transfer, using a higher value of A contributes to PCM melting and raises its rate, while at the same time, it increases E_total_. The longest total melting time and the lowest E_total_ are obtained in the case of an inner tube with a circular cross-section (A = 0). These results indicate that using a quasi-petal heat pipe, even with a low petal sinusoidal amplitude A, provides better PCM melting contribution compared to a circular tube and that increasing A improves such contribution.

**Fig 16:**
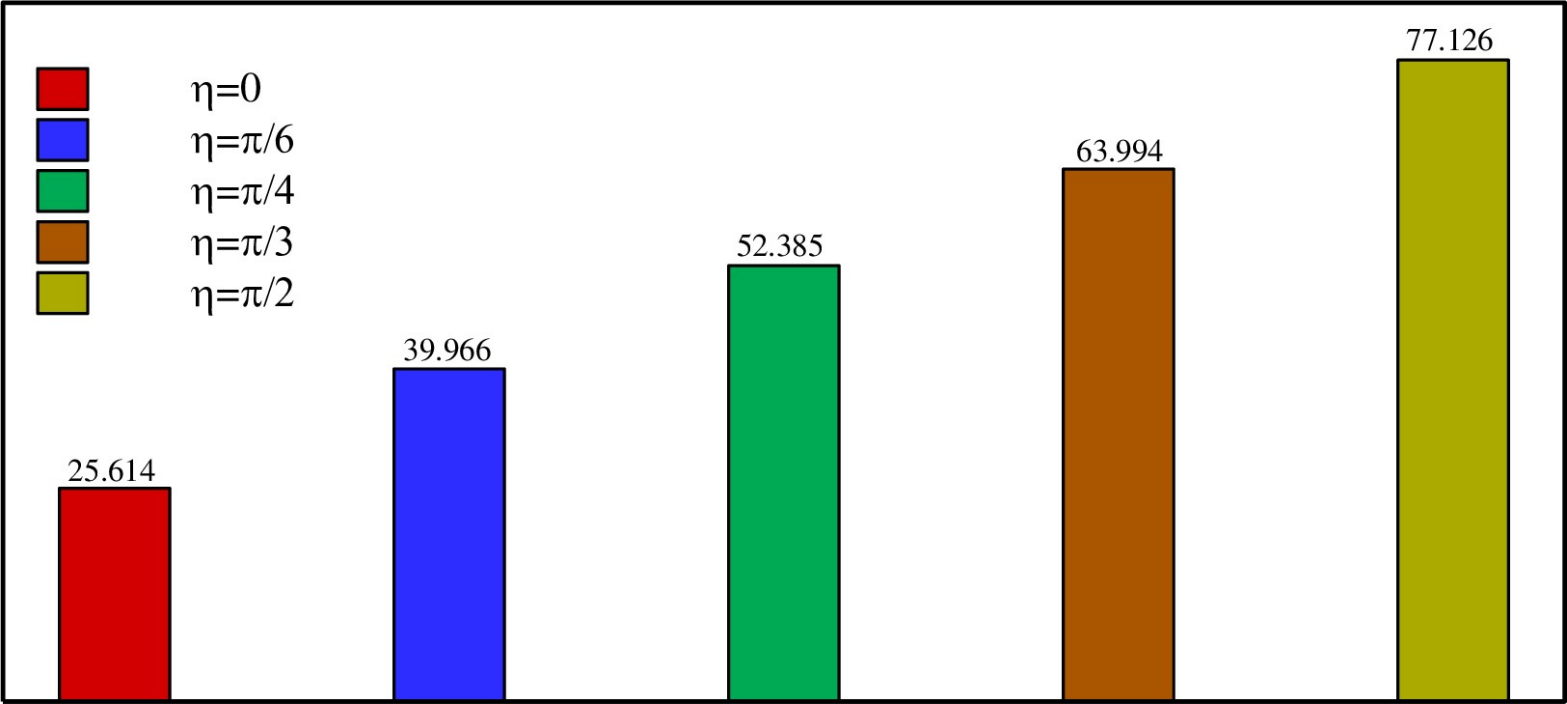
Comparison between the power (W/m) on various *η* when *r*_*d*_ = 0.6 *r*_*s*_, A = 0.15 *r*_*s*_, and Λ = 5.

**Fig 17:**
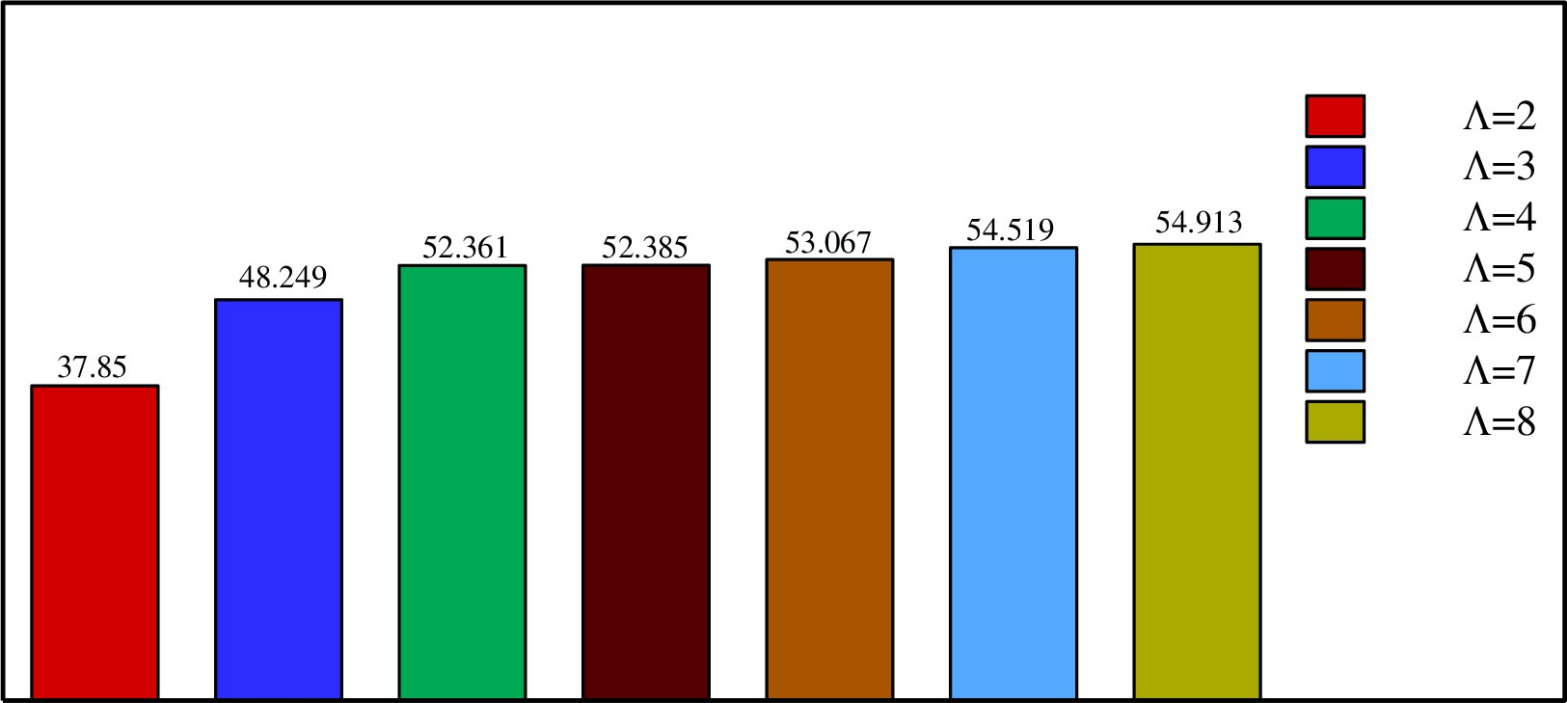
Comparison between the powers (W/m) on various the number of petals of the quasi-petal heat pipe when *r*_*d*_ = 0.6 *r*_*s*_, A = 0.15 *r*_*s*_, and η = π/4.

Figs 13–17 show a comparison between the powers for different values of η, Λ, A, and *r*_*d*_, respectively. The charging power is defined as the ratio of the total energy stored in the PCM divided by the time required to reach total melting. It is, thus, a very important parameter as it combines and summarizes the effects of the total melting time and E_total_. It can be seen from Figs 13–17 that the power is increased by raising η, Λ, A and/or *r*_*d*_. This is due, as discussed earlier, to the increase of E_total_ and the reduction of the total melting time when a higher value of η, Λ, A, and *r*_*d*_ is used. Fig 16 shows that using a vertical inner tube (η = π/2) instead of a horizontal one (η = 0) increases the power by three times. It can be seen from Fig 17 that using a tube with three petals instead of 2 raises the power by 27% while using eight petals leads to an increase of 45% compared to 2 petals. Nonetheless, only a 0.7% difference in power is obtained when using 8 petals instead of 6. Thus, Λ = 6 can be considered the optimal number of petals above which the power presents a negligible variation.

**Fig 18:**
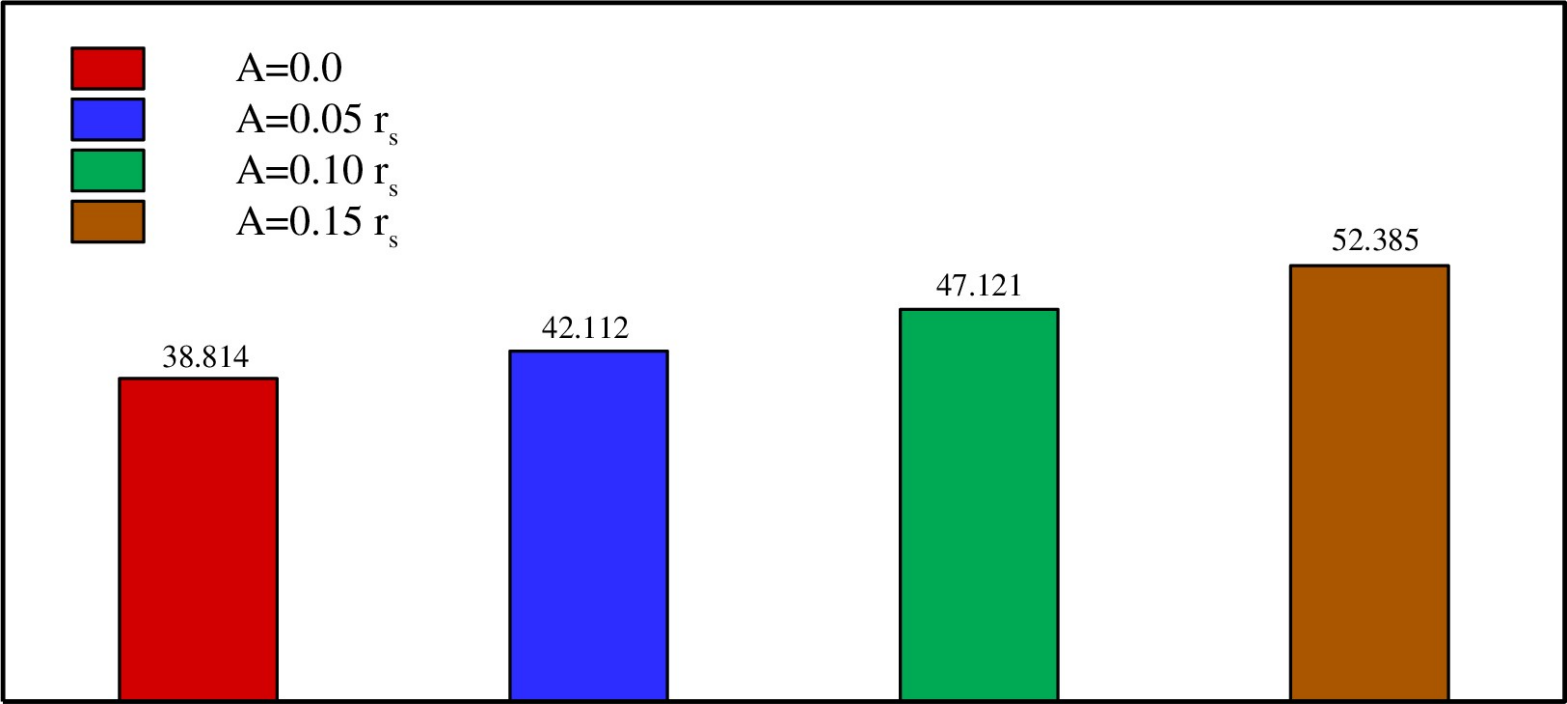
Comparison between the powers (W/m) on various the sinusoidal amplitude of the quasi-petal heat pipe when *r*_*d*_ = 0.6 *r*_*s*_, Λ = 5, and *η* = π/4.

**Fig 19:**
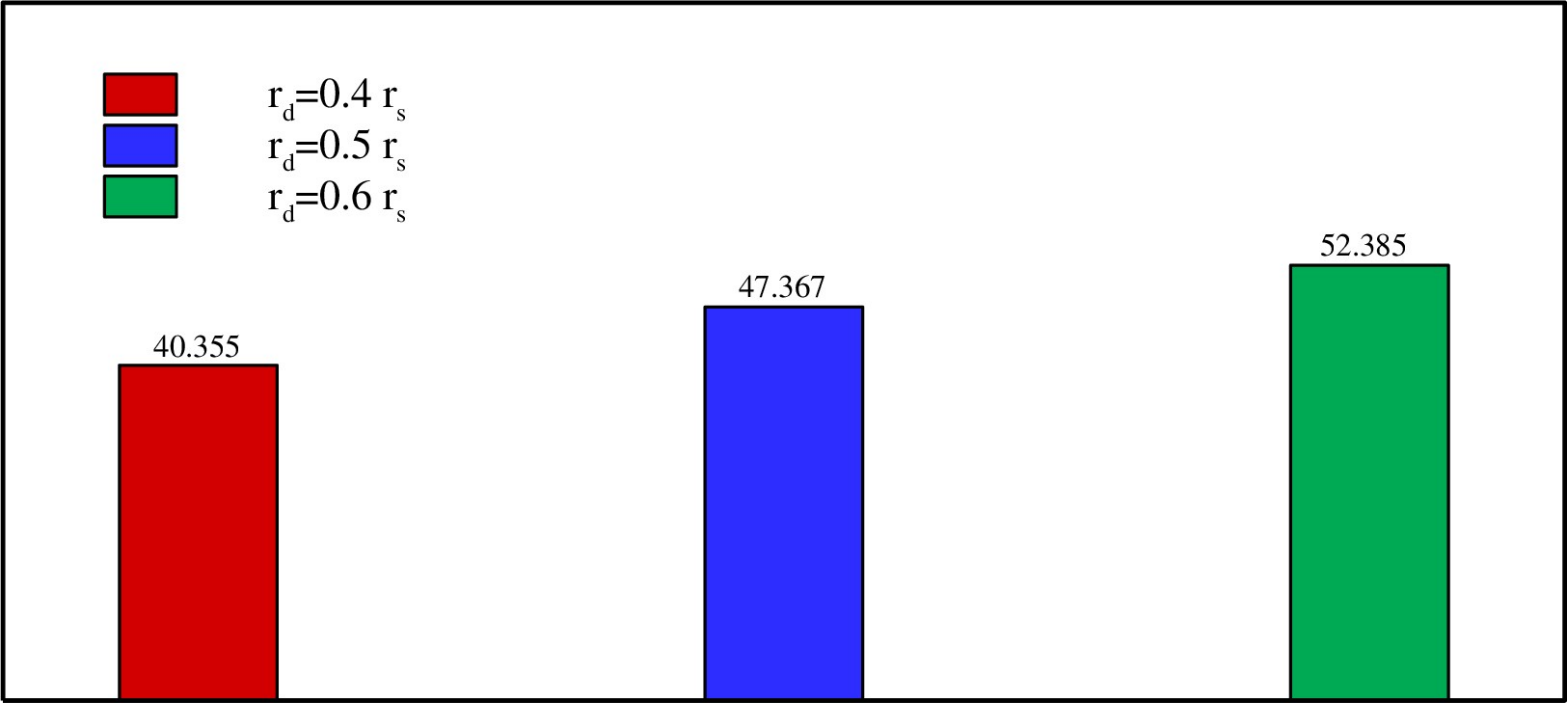
Comparison between the powers (W/m) on various *r*_*d*_ when *A* = 0.15 *r*_*s*_, Λ = 5, and *η* = π/4.

Fig 18 illustrates the fact that a quasi-petal heat pipe has more power than a circular pipe, even with a low petal amplitude. An 8% increase in the power is obtained when a petal tube with A = 0.05 r_s_ is employed instead of a circular tube with a radius r_s_. Multiplying the amplitude by 3 further increases the power by 24%. Finally, Fig 19 reveals a 30% increase in the power when the distance between the two branches of the pipe is raised 1.5 times, i.e. when the inner tube is shifted away from the center towards the shell.

## 5. Conclusion

The geometrical design of a petal foam tube for TES applications was systematically investigated. The adaptive mesh was capable of capturing the local melting interface with high resolution. The impact of petal amplitudes, petal number, the distance between the placement of tubes, and the angle of tube placements were analyzed on the melting power and temperature distribution. A summary of the main outcomes of the present investigation are presented as follows:

- Using a quasi-petal inner tube instead of a circular one increases the melting rate of the PCM and the total stored energy E_total_, due to a higher contact surface between the tube and the surrounding PCM. Moreover, increasing the number of petals, Λ, further contributes to such an increase. The charging power, defined as the ratio between E_total_ and the time required for total PCM melting, increases by 44% when a quasi-petal tube with six petals, is used instead of a circular one. Increasing Λ above 6 seems to have a negligible effect on power.
- Similar to the effect of Λ, increasing the amplitude of the petals, A, improves the contact surface between the inner tube and the surrounding PCM, and raises E_total_ while reducing the total melting time. The power shows a 24% augmentation in its value when A is increased three times.
- Reducing the distance between the two branches of the inner U-tube, r_d_, shifts the tube towards the center and limits the heat transfer and PCM melting near the outer shell, mainly in the cavity’s bottom part. Using a lower r_d_ reduces thus E_total_ and increases the time required for total melting. When r_d_ is decreased by 1.5 times, the charging power is reduced by 29%.
- The angle of inclination of the inner tube with respect to the horizontal, η, affects the heat transfer and the free convection of the melted PCM. In fact, placing the inner tube in the vertical position heats the PCM in the bottom and upper parts of the cavity and substantially enhances the convective effects. A 300% increase in the power is obtained when η is raised from 0 (horizontal position) to π/2 (vertical position).

In the current research, the geometrical impact of the HTF tube was investigated. However, the streamlines show that they reach the shell at the final stages of melting. Thus, the TES shell’s geometrical shape could also affect the natural convection flow and charging rate. The investigation of the shell design could be subject to future studies.
